# Functional insights into the infective larval stage of *Anisakis simplex s.s., Anisakis pegreffii* and their hybrids based on gene expression patterns

**DOI:** 10.1186/s12864-018-4970-9

**Published:** 2018-08-07

**Authors:** C. Llorens, S. C. Arcos, L. Robertson, R. Ramos, R. Futami, B. Soriano, S. Ciordia, M. Careche, M. González-Muñoz, Y. Jiménez-Ruiz, N. Carballeda-Sangiao, I. Moneo, J. P. Albar, M. Blaxter, A. Navas

**Affiliations:** 1 Biotechvana, Scientific Park, University of Valencia, Calle Catedrático José Beltrán 2, 46980 Paterna, Valencia Spain; 2 Departamento Biodiversidad y Biología Evolutiva, Museo Nacional de Ciencias Naturales, CSIC, Calle José Gutiérrez Abascal, 2, 28006 Madrid, Spain; 3 Unidad de Genómica, Campus de Cantoblanco, Scientific Park of Madrid, Calle Faraday, 7, Campus de Cantoblanco, 28049 Madrid, Spain; 4 Unidad de Proteomica Centro Nacional de Biotecnología, CSIC, Calle Darwin, 3, 28049 Madrid, Spain; 5 Instituto de Ciencia y Tecnología de Alimentos y Nutrición (ICTAN), Calle José Antonio Novais, 10, 28040 Madrid, Spain; 6 Servicio de Immunología, Hospital Universitario La Paz, Paseo de la Castellana, 261, 28046 Madrid, Spain; 7 Edinburgh Genomics, and Institute of Evolutionary Biology, School of Biological Sciences, The King’s Buildings, The University of Edinburgh, Edinburgh, EH9 3JT UK

**Keywords:** *Anisakis simplex*, *A. Pegreffii*, Transcriptome, Allergens, Gene expression

## Abstract

**Background:**

*Anisakis simplex* sensu stricto and *Anisakis pegreffii* are sibling species of nematodes parasitic on marine mammals. Zoonotic human infection with third stage infective larvae causes anisakiasis, a debilitating and potentially fatal disease. These 2 species show evidence of hybridisation in geographical areas where they are sympatric. How the species and their hybrids differ is still poorly understood.

**Results:**

Third stage larvae of *Anisakis simplex s.s., Anisakis pegreffii* and hybrids were sampled from *Merluccius merluccius* (Teleosti) hosts captured in waters of the FAO 27 geographical area. Specimens of each species and hybrids were distinguished with a diagnostic genetic marker (ITS). RNA was extracted from pools of 10 individuals of each taxon. Transcriptomes were generated using Illumina RNA-Seq, and assembled de novo. A joint assembly (here called merged transcriptome) of all 3 samples was also generated. The inferred transcript sets were functionally annotated and compared globally and also on subsets of secreted proteins and putative allergen families. While intermediary metabolism appeared to be typical for nematodes in the 3 evaluated taxa, their transcriptomes present strong levels of differential expression and enrichment, mainly of transcripts related to metabolic pathways and gene ontologies associated to energy metabolism and other pathways, with significant presence of excreted/secreted proteins, most of them allergens. The allergome of the 2 species and their hybrids has also been thoroughly studied; at least 74 different allergen families were identified in the transcriptomes.

**Conclusions:**

*A. simplex s.s., A. pegreffi* and their hybrids differ in gene expression patterns in the L3 stage. Strong parent-of-origin effects were observed: *A. pegreffi* alleles dominate in the expression patterns of hybrids albeit the latter, and *A. pegreffii* also display significant differences indicating that hybrids are intermediate biological entities among their parental species, and thus of outstanding interest in the study of speciation in nematodes. Analyses of differential expression based on genes coding for secreted proteins suggests that co-infections presents different repertoires of released protein to the host environment. Both species and their hybrids, share more allergen genes than previously thought and are likely to induce overlapping disease responses.

**Electronic supplementary material:**

The online version of this article (10.1186/s12864-018-4970-9) contains supplementary material, which is available to authorized users.

## Background

*Anisakis* is a genus of nematodes (Nematoda, Rhabditida, Ascaridomorpha: Anisakidae [1]) which parasitizes marine mammals, fishes, molluscs and crustaceans. *Anisakis* nematodes complete their life cycle in the stomachs of cetaceans, and less frequently pinnipeds, which become infected after devouring paratenic hosts (fishes, cephalopod molluscs or krill) harboring the arrested infective third-stage larvae (L3) [2]. Humans can also be infected by consuming raw or undercooked fish or cephalopod meat. Although L3 of *Anisakis spp* larvae are unable to complete their life cycle in humans, after penetration of the human gastrointestinal tract they can cause anisakiasis, a condition characterized by acute gastrointestinal manifestations of epigastralgia, nausea, abdominal pain and diarrhoea [3]. Allergic reactions such as IgE-mediated hypersensitivity, angioedema, urticaria and anaphylaxis are also associated with zoonotic exposure of humans to *Anisakis* spp. [4]. Fish infected with L3 are treated by freezing, which kills the larvae. However, while infection by live L3 is necessary for many of the symptoms of anisakiasis, humans sensitized to *Anisakis* may show symptoms after consuming fish that has been correctly frozen, cooked and even processed [5–7]. Many *Anisakis* spp. proteins maintain their allergenic properties even after thermal treatment [7–9]. Episodes of allergic disease in the absence of recent allergen exposure have also been also described [10].

Anisakiasis is emerging as an important epidemiological problem. More than 20,000 cases of anisakiasis have been reported worldwide since 1960 [11], with higher incidences in areas such as Japan, the Netherlands, France, Spain, Germany and California where raw fish is traditionally or increasingly being eaten. Given the widespread consumption of raw fish in the form of sushi and sashimi, Japan has however the highest prevalence of gastric anisakiasis. In Spain, where *Anisakis* spp. are considered to be an emerging health problem [6], infection frequency ranks between 0.43 and 22% in fish that reach the market have been reported, with very high rates of infection in particular species [12].

The allergenic potential of *Anisakis* spp. resides in their allergome (or full set of encoded allergen products). *Anisakis* spp. contains at least 15 distinct types of major allergens (Ani s 1 to Ani s 14 and Ani s 11.0201) according to the Allergen Nomenclature site (http://www.allergen.org) [13, 14]*.* Up to 28 different proteins described as potential new allergens were identified by proteomic analysis of *A. simplex s.s.* and *A. pegreffii* [15]. A transcriptomic analysis reported 36 and 39 allergens in *A. simplex s.s.* and *A. pegreffii* respectively [16]*.*

Morphological taxonomy of *Anisakis* is based on excretory system and esophageal intestinal region of L3 [17, 18] and the morphology of adult males [19]. As sampling of adult *Anisakis* from within definitive hosts is problematic, and L3 diagnosis is difficult, a molecular taxonomic approach for L3, using the nuclear ribosomal internal transcribed spacers (ITS), has been developed [20]. These markers have revealed both potential additional species diversity within *Anisakis* as well as recombinant genotypes [21–23]. The genus *Anisakis* is commonly accepted to include 10 named species: *A. simplex* sensu stricto (*s.s.*), *A. berlandi* (formerly *A. simplex C*), *A. pegreffii*, *A. typica*, *A. ziphidarum*, *A. physeteris*, *A. brevispiculata*, *A. paggiae*, *A. nascettii* and *A. schupakovi*. At least 2 additional undescribed species (*Anisakis* sp. 1 and *Anisakis* sp. 2) have also been designated from L3 genotypes [24, 25]. The most widely studied species are *A. simplex s.s.*, *A. pegreffii* and *A. berlandi*: these constitute the *Anisakis simplex* sensu *lato* (*s.l.*) complex [26–28]. *A. simplex s.s.* and *A. pegreffii* have been identified as the main etiological agents of anisakiasis [29–33] but it remains unclear if these 2 species are equally pathogenic.

Importantly, *A. simplex s.s.* and *A. pegreffii* are able of hybridizing in the areas where they are geographically sympatric [34, 35] and co-infect the same fish host [36]. The described “species” with recombinant genotypes may be the products of interspecific hybridization [34]. While no fertile adult hybrids have been found, L3 with genotypes suggestive of hybridisation between *A. simplex s.s.* and *A. pegreffii* have been identified [24]. These hybrids may express distinct suites of allergenic and immunoreactive proteins [15].

Here we present a transcriptomic analysis of *A. simplex s.s., A. pegreffii* and their hybrids sampled in the same development stage (L3) from the same host (*Merluccius merluccius*) captured in waters where *A. simplex s.s.* and *A. pegreffii* are sympatric. We used these data to compare their metabolic profiles, gene ontology profiles and expression patterns. We also present an online database, AnisakisDB, which provides access to the abundant data we have generated.

## Methods

### Taxonomic identification and selection of specimens for RNA sequencing

Larvae specimens in the same development stage (third stage larvae L3) of *Anisakis* were obtained from the FAO 27 area of fishing distribution into a general survey carried out by our laboratory. L3 were extracted from hosts following published procedures [34, 37, 38]. L3 were rinsed in 0.9% saline solution, placed in an antibiotic solution for 30 min and then rinsed in bi-distilled water for 1 h before molecular identification and RNA extraction. The larvae were individualized (from each host) and for each specimen the caudal part was used for DNA extraction and PCR amplification for species identification; this caudal part and the rest of the body were separately stored at -80 °C until required. Species identification in that survey was performed following the taxonomic criteria of [23, 39] by using the ITS1 region of the nuclear ribosomal DNA (rDNA). Breifly: individual *Anisakis spp.* L3 stage juveniles was placed in an Eppendorf tube after previously having a small part of the caudal region removed to allow molecular identification of each individual. DNA was extracted and purified using the Speedtools Tissue DNA Extraction Kit (Biotools) following manufactures instructions. Molecular identification was carried out for each individual using PCR-RFLP. The forward primer A 5’-GTCGAATTCGTAGGTGAACCTGCGGAAGGATCA-3′ and reverse primer B 5’-GCCGGATCCGAATCCTGGTTAGTTTCTTTTCCT -3′ [39] were used in reactions containing, 10 mm Tris-HCl (pH 8.3), 1.5 mm MgCl2, 50 mm KCl, 200 mm each of dATP, dCTP, dGTP and dTTP and 1 unit of DNA polymerase (Biotools B and M labs, S.A. Madrid, Spain). Initial denaturalization was carried out for a period of 2 min at 94 °C followed by 35 cycles of 94 °C for 1 min, 58 °C for 1 min, 72 °C for 1 min followed by a final 7 min extension at 72 °C. Amplified DNA fragments were digested with the restriction enzymes HhaI and HinfI (New England Biolabs, Massachusetts, MA, USA) following manufacturer’s instructions. Restriction fragments were separated by electrophoresis in Tris- Borate-EDTA (TBE) buffered 2.5% agarose gel, stained with SYBERsafe and visualized with UV illumination.

#### Specimens selection for trancriptomics

The detected hybrids can differ as the species (*A. simplex* s.s. or *A. pegreffii*) contributing the maternal and paternal genomes, which can influence their expression patterns. We have used mitochondrial DNA sequences, which are inherited maternally, to differentiate the two types of hybrids. We obtained partial sequences of the mitochondrial cytochrome oxidase subunit 2 (COII) gene [40] from each hybrid and determined its maternal species by comparing the COII sequence with a set of COII sequences obtained from 100 populations of *Anisakis*, using molecular phylogenetic methods. The COII gene was amplified as detailed in [40]. The obtained sequences were pooled with 41 other *Anisakis* COII sequences downloaded from GenBank [41], and then aligned using the ClustalW program [42] as implemented in Bioedit [43]. Phylogenetic relationship of populations was obtained by Maximum Likelihood and Bayesian Tree (Evolution Model: GTR + I + G) [44] (Additional file 1) considering as outgroup the sequences of *Contracaecum* sp. and *Toxocara canis*. The Bayesian information criterion (BIC) as implemented in jModelTest v2.1.4 [45] selected GTR + I + G (*I* = 0.4950; G = 0.6230) as the evolutionary model that best fit the data. The selected model and model parameters were used in the maximum likelihood (ML) analysis performed with PhyML v.3.1 [46]. The robustness of the inferred trees was tested by nonparametric bootstrapping (BP) using 1000 pseudo replicates. Bayesian inference was also performed with MrBayes v.3.2.5 [44], running for 1 × 10^7^ generations (four simultaneous Markov chains; sample frequency 100). Four independent partitioned analyses were performed and checked for stationarity and convergence of the chains with Tracer v1.6 [47]. Three data partitions were analyzed: first, second, and third codon positions. Model parameters were estimated independently for each one of the respective data partitions. Burn-in was set to the first 1.000.000 generations. The robustness of the inferred Bayesian trees was determined using Bayesian posterior probabilities (BPP; as obtained from majority-rule consensus trees of the post burn-in trees) (provided as Additional file 1).

In order to minimize a possible host effect, the larvae used for the three RNA sequencing experiment were obtained from ten different individuals of the same fish host (European hake, *Merluccius merluccius*) and when it was possible, from fishes which were parasited at the same time by A*. simplex s.s., A. pegreffii* and their hybrid haplotypes (labeled by yellow and green colors in Additional file 2).

RNA samples from the 3 groups of selected specimens were pooled and 500 ng of each processed using the Illumina TruSeq RNA sample prep kit v.2 (Illumina) to generate 2 poly(A)-selected libraries, one with small inserts (SI library; size range = 200–325 bp) and one with large inserts (LI library; size range = 260–840). Paired-end reads (2 × 100 bases) were generated on Illumina HiSeq 2000. A total of 19–23 million pass-filter read pairs were obtained from each library. Raw reads were converted to fastq format using Casava on the BaseSpace platform [48]. Read set qualities were assessed using FastQC [49], and trimmed of adapters and low quality data with Cutadapt [50] and Prinseq [51]. An assembly was produced independently for each species (*A. simplex s.s.* and *A. pegreffii*) and for the hybrids. The SI and LI libraries trimmed fastq files from each specimen group were assembled de novo using Oases [52, 53] using a kmer range of 21–31 and following the specifications indicated in the Oases manual. All the assembly metrics provided in Table 1 were obtained using the Perl script Assemblathon stats [54] with the exception of the number of unigenes, which was provided by Oases.

**Table 1:** Metrics for de novo assembly transcriptomes

Summary	*A. simplex s.s*	*A. pegreffii*	Hybrid	Merged
Total transcriptome size	88,007,524	68,071,234	50,568,936	67,459,080
Unigenes (Loci)	36,645	31,988	29,656	-NA-
Transcripts (isoforms)	121,907	91,541	76,848	75,380
Longest transcript (bp)	10,774	11,724	10,798	16,240
shortest transcript (bp)	100	100	100	107
% transcripts >1Kb	20.3%	21.7%	17.9%	28.7%
N50 (bp)	973	1026	885	1276
L50 (bp)	25,878	19,118	16,598	15,385
%A	30.87%	30.25%	30.11%	30.41%
%C	19.60%	20.03%	20.36%	18.64%
%G	19.64%	19.98%	20.49%	20.60%
%T	29.89%	29.74%	29.04%	29.87%
%Ns	0%	0%	0%	0%

### Sequence annotations

The transcriptomes of *A. simplex s.s., A. pegreffii* and the hybrids were annotated using the NCBI tool blastx [55] with an evalue of 10e-^5^ as cutoff threshold and the NCBI non-redundant protein (NR) and the Eukaryotic Orthologous Groups (KOG) databases [56] as reference subjects. Gene Ontology (GO) terms as well as their evidence code [57] and Enzyme Commission (EC) numbers [58] were assigned to annotations using correlation tables among GenBank accessions and GO and EC information. InterPro domain [59] annotations were also annotated using identical methodology. Transcripts that had no informative blastx matches were further used to search the NCBI nucleotide database (NT) using NCBI BLAST tool blastn and identical evalue cut-off. The annotation protocol is automatized as a pipeline cited in the section below “pipelines”. Proteins (open reading frames) longer than 75 residues were predicted from transcripts using Transeq from the EMBOSS package [60] and OrfPredictor [61]. Signal 4.1 [62] and TMHMM v.2.0 [63] were used to predict secretory signal peptides and transmembrane regions. Metabolic pathways were retrieved from the KEGG web site [64] using EC annotations as queries.

### Reference transcriptome

Minimus2 from the Amos package [65] was used to merge the 3 assemblies into a merged transcriptome. Remaining redundancy was eliminated using CD-HIT [66] at a similarity threshold of 0.95. The merged transcriptome after redundancy filter resulted in 75,380 consensus transcripts that were annotated via BLAST as described above for transcriptomes using the NR database.

### Differential expression and enrichment analyses

Bowtie2 [67] was used to map the two RNAseq pair-end libraries sequenced per each *Anisakis* sample on the merged transcriptome. In all cases over 97% of the reads were successfully mapped on the reference (data not shown). Next, Corset [68] was used to parse the bam files resulting from the mapping step and extract a cluster file grouping the 75,380 consensus transcripts into 74,751 transcript clusters and a count file summarizing the read counts obtained per each cluster from all pair-end libraries mapped to the reference merged transcriptome. The count file was used as input to EdgeR [69] to perform 3 differential expression tests at the whole-transcriptome level: “hybrids vs. *A. pegreffii*”, “hybrids vs. *A. simplex s.s*.” and “*A. pegreffii* vs. *A. simplex s.s.*”. The SI and LI pair-end libraries prepared from each sample were considered as technical replicates. Transcripts were considered as differentially expressed when they had a *p*-value < 0.05 after False Discovery Rate (FDR) correction applied by EdgeR using the Benjamini-Hochberg method [70] (referred as just FDR through the rest of the manuscript). Transcripts clusters were assigned the annotations of their consensus transcripts using the GPRO worksheet [71]. Clusters from transcripts with no NR/NT annotations were considered in differential expression analyses only if they had non-zero read counts in at least 2 of the 3 samples.

GO and metabolic pathway enrichment analyses were performed using GOseq [72] and the results from differential expression as input, following the GOseq manual indications for non-native transcriptomes. Concretely, we used 4 input files: 1) a file summarizing the consensus transcripts found as differentially expressed as a FDR < 0.05 as a cutoff in differential expression analyses; 2) a file with all assayed consensus transcripts; 3) a file with the GOs or with the metabolic maps associated to the consensus transcripts (depending on the analysis); and 4) a file with the sequence size of each assayed cosensus transcript. A priori, enriched GO terms and metabolic pathways supported by *P*-values < 0.05 in the resulting Wallenius distribution, were considered as significant. A 5% FDR correction was performed on the P-values obtained from the enrichment analyses using the Benjamini-Hochberg method to outline the most enriched GOs and pathways.

With the aim to also asses differential expression of genes encoding for excreted or secreted (ES) compounds read counts of transcript clusters with annotation of secretory leader peptide from Signal-P domain or with annotation of GO terms for extracellular compartments were exported to a new count file. Counts in this file from transcripts sharing the same protein descriptions (from NR annotations) were combined using a custom php script called combine_counts.php that is available in AnisakisDB (see the section below AnisakisDB). We combined information from the Uniprot browser [73] and the literature to filter the transcript set to include only those verified as coding for ES products or with confirmed secretory signal peptides but unknown sub-localization. The final count file had 356 non-redundant protein descriptions associated with genes encoding putative ES products. Three differential expression analyses (“hybrids vs. *A. pegreffii*”, “hybrids vs. *A. simplex s.s*.” and “*A. pegreffii* vs. *A. simplex s.s.*”) were performed using EdgeR, with the small and large insert size libraries of each transcriptome considered as technical replicates (as above).

A third differential expression study focusing on the distinct families of allergens detected in the transcriptomes of *A. simplex s.s., A. pegreffii* and the hybrids, was also performed. We retrived 509 allergen sequences belonging to 150 allergen families (AllFams) in fungi, plants and animals from the Uniprot protein knowledgebase [73]. We have also used additional information provided by the AllFam database of allergen families [14], and the WHO/IUIS Allergen Nomenclature Database [13, 14]. These 509 allergen sequences were used to identify (via blastx search) homologs in the merged transcriptome. Nine hundred and thirty seven consensus transcript were identified as potential allergen candidates based on a blast e-value threshold of 2e^− 06^. Using identical methods to those described above for ES protein genes, read counts for these 937 candidates were collated for each AllFam family of allergens in a count file that was then used as input to perform 3 differential expression analyses using EdgeR as above.

### Pipelines

The above described protocols for pre-processing, de novo assembly, annotation and differential expression were executed using the DeNovoSeq and the RNAseq pipelines provided by the GPRO suite [70].

### AnisakisDB

**Fig. 1:**
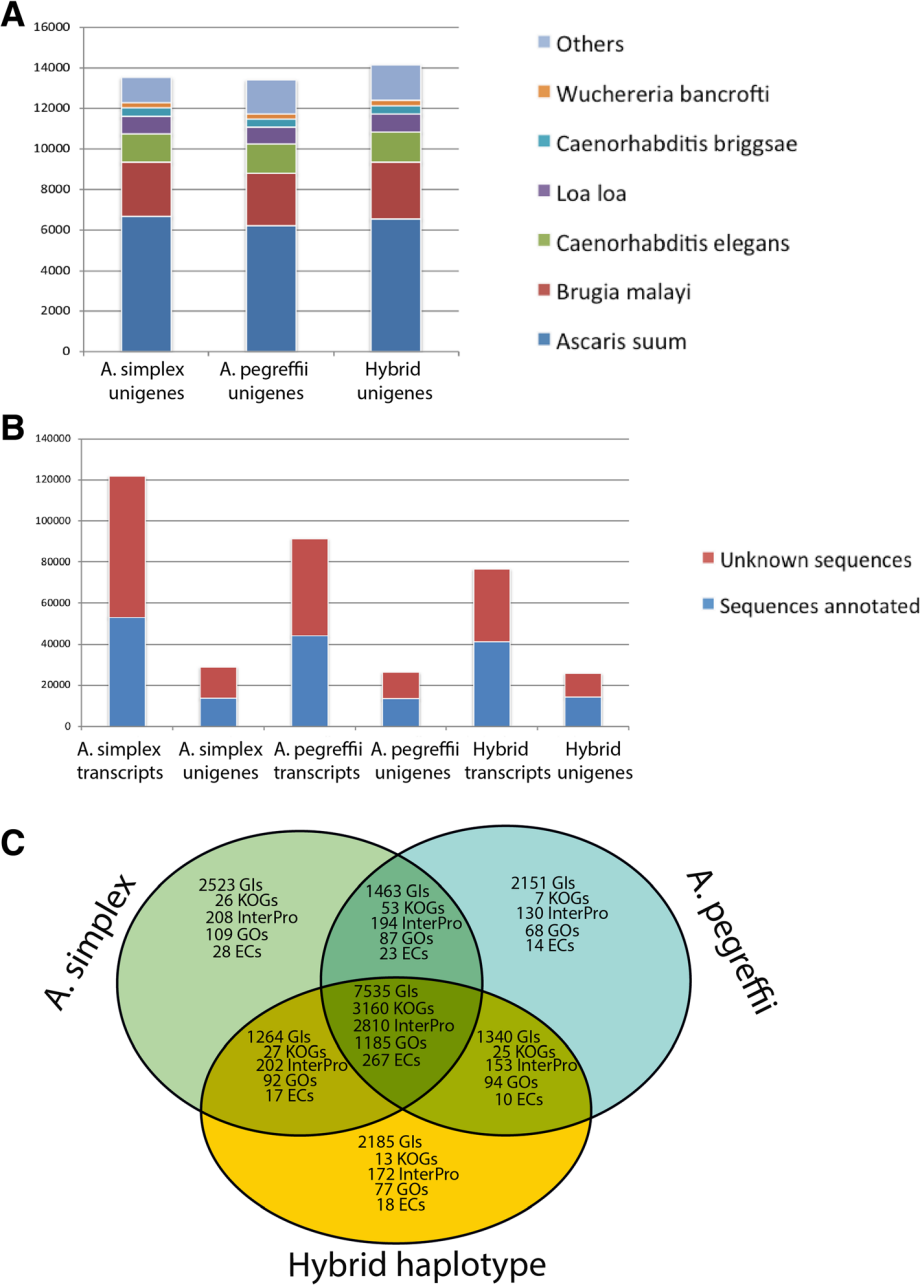
Annotations. **a** Blast top hits per subject species using NR as refseq database for unigene annotation. **b** Distribution of annotated and un-annotated transcripts and unigenes annotated via blast using NR as refseq database. **c** Venn diagram showing unique and shared features of non-redundant annotations per transcriptome (GIs, KOGs, InterPros, GOs, ECs)

We have reconstructed the transcriptomes of the L3 species *A. simplex s.s.* and *A. pegreffii* and their hybrids producing a collection of 290,296 transcripts spanning more than 200 Mb. We have also reconstructed a consensus transcriptome of 75,380 consensus sequences. Sequences have been annotated using different databases and classificatory systems (NR/NT, KOG, InterPro, GO, EC) thus creating abundant material (annotations and other material such as scripts, statistics graphical representations and the differential expression and enrichment files). To house the aforesaid material and make it freely available for readers, we constructed a web-available database, AnisakisDB. The database was programmed using Laravel 5 framework for public client platform and a CodeIgniter 2 framework for server platform, both based on the PHP programming language [74], a MySQL database [75] and an Apache server [76] hosted in a Linux environment. The database portal includes a blast search powered by the NCBI BLAST package [77]. Within AnisakisDB, sequences, annotations and backups of all performed analyses of differential expression and enrichment are distributed in seven sections (“Transcriptomes”, “Blast”, “Download”, “COII markers”, “Secretome” and Allergome) managed by an intuitive menu and additional utilities to retrieve or compare sequences and annotations. In addition, within the section “Transcriptomes” we have implemented different web site sections providing access to 5 independent Venn Diagrams created based on the GI, KOG, InterPro, GO, EC annotations used to create the Venn diagram of Fig. 1. In each Venn-based web site, clicking on any intersected number the Venn opens a dialog summarizing the annotations that correspond to the intersection. The Section “COII markers” is another web site where the COII-based phylogeny inferred for selection of specimens has been graphically implemented, also as a dynamic tree representation. AnisakisDB is freely and online accessible at www.anisakis.mncn.csic.es.

## Results and discussion

### Transcriptome de novo assembly and annotation

We sampled *Anisakis* L3 from a single ocean region (FAO 27) and fish species (*M. merluccius*), to avoid issues that might arise from response to different paratenic hosts and minimize other environmental and phylogeographic confounding factors. L3 were identified according the taxonomic approaches of [23, 39] by using the ITS1 region from rDNA. The phylogenetic tree of 141 COII sequences from 100 populations plus another 41 sequences obtained from GenBank showed all *A. simplex s.s* and all *A. pegreffii* samples clustering in separate sister clades (Additional file 1). The tree also showed that all COII sequences from hybrids fell into one or another of these two clades, allowing immediate classification of hybrids as to their maternal species. RNA was extracted from pools of 10 *A. simplex s.s.* 10 *A. pegreffi* and 10 hybrids (five mothered by *A. simplex s.s.* and five mothered by *A. pegreffii*). Two paired-end RNA-Seq datasets were generated from each pooled sample, forming technical replicates of the transcriptome. Four transcriptome assemblies were generated, one for each sample (*A. simplex s.s., A. pegreffii* and hybrids) and one that merged the 3 sample-derived ones (Table 1). The 3 sample-derived transcriptomes are similar in span and complexity. The assembly of *A. simplex s.s.* was longer and had an elevated number of predicted transcripts, apparently because of a greater number of isoforms (30,366 more than *A. pegreffii* assembly and 45,059 more than the hybrid assembly). We think this might be due to sequencing technical issues, elevated levels of alternative splicing (i.e. isoforms) or elevated heterozygosity in the transcriptome of *A. simplex s.s.* as the number of unigenes is in contrast quite equilibrated (4657 unigenes more than *A. pegreffii* assembly and 6989 more than the hybrid assembly).

#### Sequence accessions

COII sequences have been deposited at European Nucleotide Archive (ENA) under the accession numbers LT883269 - LT883368 (Additional file 2) (For more details, see the section in results Introducing AnisakisDB). Raw read data have been deposited at the NCBI SRA Study SRP072976, BioProject PRJNA316941 and BioSamples SAMN04592605, SAMN04592630, and SAMN04592599.

The transcriptomes of *A. simplex s.s., A. pegreffii* and hybrids were annotated by sequence similarity via blast searches performed against the protein and nucleotide NR, KOG and NT NCBI databases considering an e-value threshold cutoff of 10e-5 and via prediction of secretory signal peptide and transmembrane regions. Annotation features (InterPro domains, GOs, and ECs) were also retrieved from the annotations of protein blast subjects provided by NR database. All annotations are summarized in Table 2. The best hits detected per species in the blast search performed against NR database were provided by proteins of other nematodes such as *Ascaris suum, Brugia malayi,* and *Caernorhabditis elegans* (Fig. 1a). Although the transcriptome of *A. simplex s.s*. had the highest number of transcripts, most of these did not detect homologues via blast (Fig. 1b). As it is shown in Table 2, if considering only transcripts with annotation from NR/NT databases, the 3 transcriptomes result in similar number of annotated transcripts (assembled isoforms) and unigenes (loci to which a set of transcripts is assigned). Considering the Gene Identifiers (GIs) to approximate the number of expressed genes we can state that the reconstructed transcriptomes of *A. simplex s.s., A. pegreffii* and the hybrids were annotated based on 12,785, 12,489 and 12,324 potential genes, respectively. Annotations thus correct the bias in number of isoforms detected in the assembly of the *A. simplex s.s* transcriptome, suggesting these sequences might be unknown sequences or potential sequencing artefacts (which for simplicity’s sake were not considered in the subsequent comparative analyses). When considering the annotations of the three *Anisakis* transcriptomes altogether a total number of 18,461 non-redundant GIs were found, which approximate the annotated pan-transcriptome here characterized from A*.simplex s.s., A. pegreffii* and their hybrids. Of the 18,461 total GIs, the 3 transcriptomes share 7535 GIs providing additional functional information in the form of 3260 KOG annotations, 2810 InterPro domain annotations, 1185 GO categories and 267 ECs (Fig. 1c). As also shown in that figure, the reconstructed transcriptome of *A. simplex s.s.* shares 1463 GIs with *A. pegreffii* and 1264 GIs with the hybrids. In turn, the hybrids share 1340 GIs with *A. pegreffii.* This therefore suggests that (based on the annotations of these 3 transcriptomes) the hybrid shares at least 8875 GIs with *A. pegreffii* and 8799 GIs with *A. simplex s.s.* In other words, at least 48 and 47.6% of the annotations of the hybrid transcriptome have homologs in A. *pegreffii* and A. simplex s.s., respectively (consistently with the hybrid status). *A. simplex s.s* shares 8998 GIs with A*. pegreffii* thus meaning that at least 48.7% of the unigenes annotated in *A. simplex s.s* have homologs in *A. pegreffii*.

**Table 2:** Summary of annotations per transcriptome

Sequences with annotations	*A. simplex s.s.*		*A. pegreffii*		Hybrids	
	transcripts	unigenes	transcripts	unigenes	transcripts	unigenes
Gene Identifiers (GIs) from NR/NT	62,483	14,057	60,816	14,393	53,741	14,183
KOG clusters	38,717	9999	29,977	9444	28,114	9928
InterPro domains	17,093	5938	13,514	5122	12,884	5125
GO Terms	14,701	5165	11,773	4485	11,101	4501
ECs	5416	1995	4237	1671	3913	1617
Predicted proteins	49,755	14,384	41,324	13,506	38,782	13,834
Predicted signal-P domains	1124	647	603	394	482	318
Predicted TM domains	11,613	6338	8906	4676	7545	4109

### Expression patterns and enrichment of metabolic pathways and gene ontologies

**Fig. 2:**
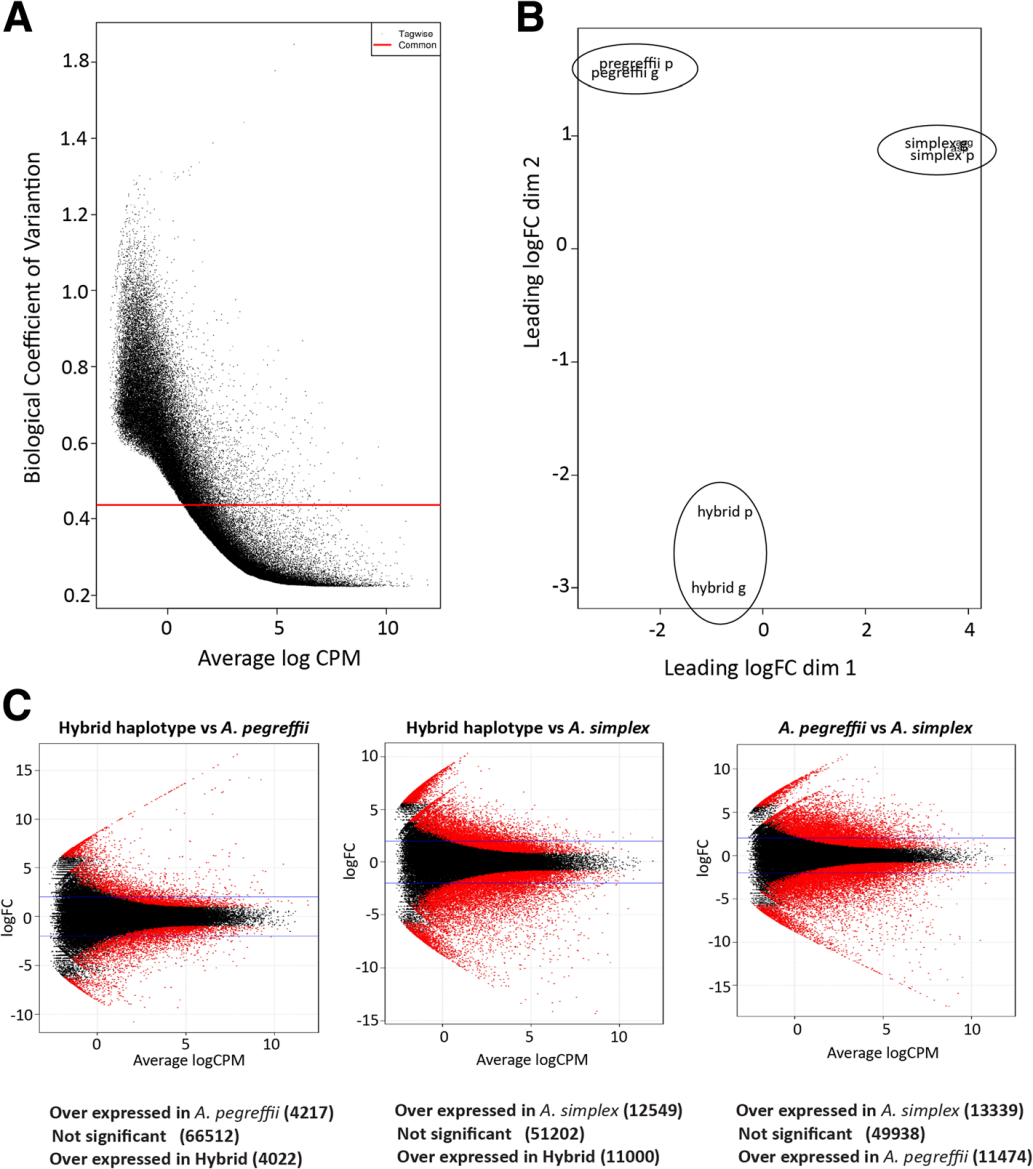
Transcriptome differential expression patterns. **a** BCV versus the average log of counts per million (CPM). **b** MDS plot based on logFC of differences among taxa sources and the sequenced replicates per taxa (small and medium insert size fastqs). **c** MA-plots (one per each differential expression analysis) representing the log Fold Change (logFC) against the log-average log CPM per each transcript cluster across each pair of compared samples. Differentially expressed clusters supported by FDR < 0.05 are plotted in red. Each MA-plot is accompanied by a summarization of the results of each differential expression comparison

The merged (consensus) transcriptome reconstructed in this study based on the three *Anisakis* transcriptome consist of 75,380 consensus sequences, which were used as a mapping reference to compare the expression patterns at the whole-transcriptome level of the 3 taxa. Of the 75,380 consensus transcripts, 32,999 have no annotation and therefore correspond to unknown sequences expressed by the at least two of three taxa (they might be unknown proteins, potential non-coding RNAs and/or even mobile elements). The remaining 42,381 consensus transcripts correspond to coding sequences annotated based on 12,511 different proteins (according to the number of non redundant GI annotations) thus meaning that any biological interpretation of differential expression and enrichment based on the merged transcriptome is based on the expression patterns of 12,511 coding genes (around 68% of the pan-transcriptome here characterized based on the three transcriptomes). After mapping the reference transcriptome, the 75,380 consensus sequences of the merged transcriptome were grouped into 74,751 transcript clusters (see Methods) correcting any potential bias in isoforms as detected in the assembly of *A. simplex s.s*. The Biological Coefficient of Variation (BCV) and the average dispersion of the assayed samples were inferred and evaluated to asses to suitability of the fastq libraries, resulting in quite appropriate values (BCV = 0.4359 and dispersion = 0.19005) for a study of differential expression. A similar conclusion resulted from plotting BCV against the average log of counts per million of mapped reads. Additionally, a multidimensional scaling (MDS) plot based on logFC of the differences among taxa sources and the sequenced replicates per taxa shows that while the SI and LI libraries used as taxon replicates fall close to one another, the replicates of different taxa fall widely separated (Fig. 2b). This result anticipates important differences among species and hybrids. The first plot dimension correspond to the differences among taxa sources (*A. simplex s.s.*, Hybrids, *A. pegreffii*) that provides the major source of variability. The second plot dimension corresponds to the sequenced replicates per taxa (small and medium insert size fastqs) that present practically no differences with the exception of the hybrids where the 2 replicates are slightly more heterogeneous because they were created as a pool of 5 hybrids close to *A. simplex s.s.*, and 5 other close to *A. pegreffii*. Consistent with this perspective, of these 74,751 transcript clusters, 8239 resulted as differentially expressed in the analysis performed between the hybrids and *A. pegreffii* using FDR < 0.05 as cutoff, 23,549 were differentially expressed in the analysis performed between the hybrids and *A. simplex s.s.* using same FDR, and 24,813 were differentially expressed between *A. pegreffii* and *A. simplex s.s.* with same FDR (Fig. 2a and Additional file 3). In general, these analyses of differential expression based on the merged transcriptome suggest that the *A. pegreffii* parental expression pattern predominates over *A. simplex s.s.* in the hybrids, in agreement with population genetic studies of Moroccan *Anisakis* [36].

**Fig. 3:**
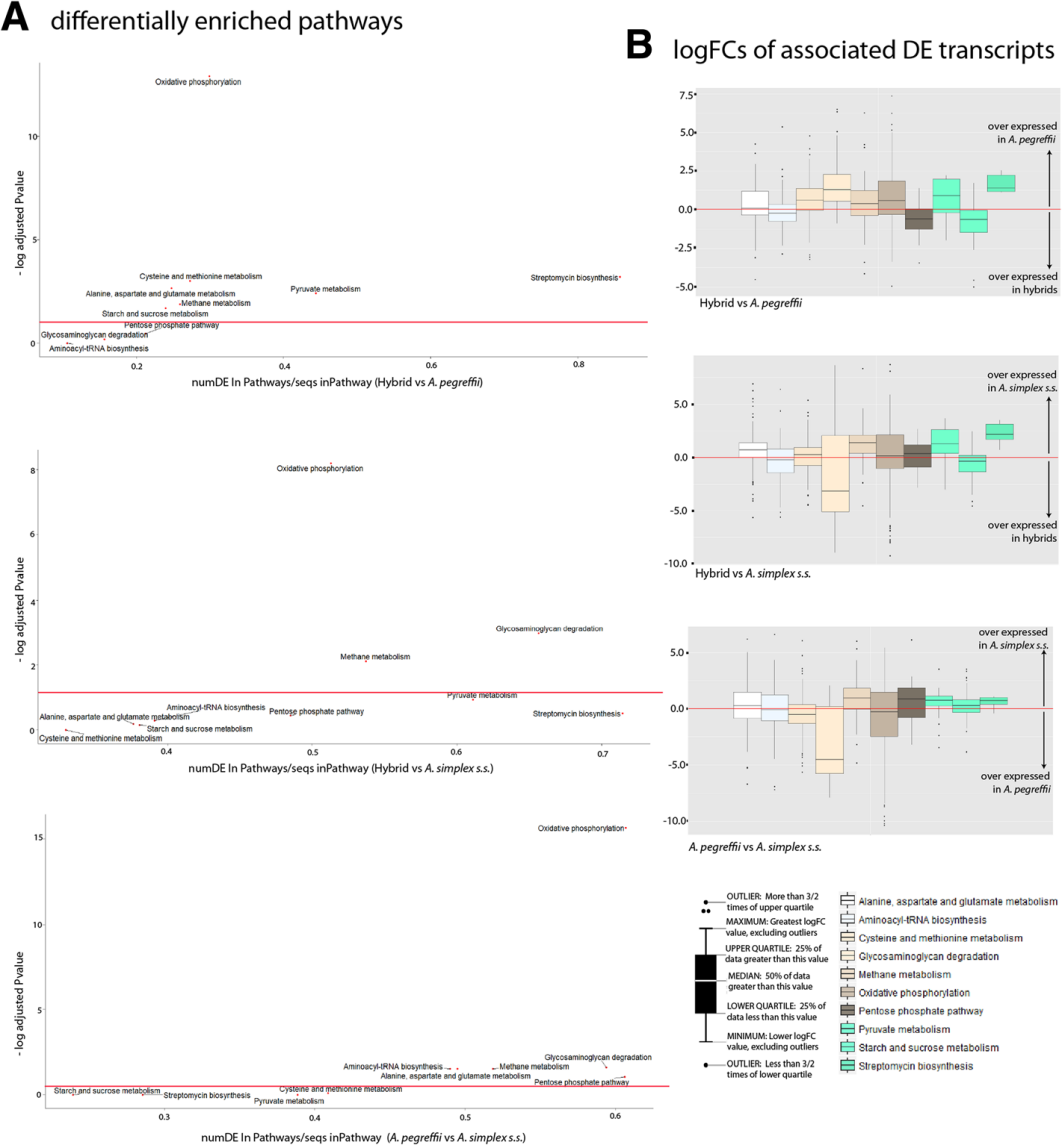
Enrichment of metabolic pathways. **a** Scatter plot for the enrichment of metabolic pathways per differential enrichment comparison at FDR < 0.05. In the x-axis, the ratio between number of DE transcripts and total number of transcripts per pathway using the Wallenius approximation; in the y-axis, the log *P*-value for differential enrichment in each pathway. **b** Box plots based on the median, quartiles, maximum and minimum and outlier values of the LogFCs of all transcripts contributing to the enrichment of the metabolic pathways shown in A in each differential expression comparison. Red lines highlight the logFC value of zero

The 272 ECs identified as common in the 3 transcriptomes link to 53 metabolic pathways [64] related to nutritional requirements and biosynthesis, energy metabolism, metabolism of xenobiotics, environmental information processing, signal transduction and genetic information processing. Ten of these 53 pathways were shown as differentially enriched in at least one of the 3 enrichment comparisons after correction of the pvalue at FDR < 0.05 (Additional file 4). As shown in Fig. 3, two of these pathways (oxidative phosphorylation and methane metabolism) were differentially enriched in all comparisons thus indicating that the main metabolic differences among A*. simplex s.s., A. pegreffii* and their hybrids concern to the energy metabolism. Both *A. pegreffii* and *A. simplex s.s.* present overexpression with respect to the hybrids as for transcripts associated to methane metabolism while differentially expressed transcripts involved in oxidative phosphorylation are overexpressed in *A. pegreffii* with respect to the 2 other taxa. Figure 3 also reveals that the enrichment of the glycosaminoglycan degradation pathway was significant between the performed comparisons of the hybrids and *A. pegreffii* against *A. simplex s.s.* but not when the hybrids and *A. pegreffii* are compared. The degradation of glycosaminoglycans pathway mainly concerns to transcripts coding for hyalurononglucosaminidases, which are known to play role in the extracellular matrix as soluble components and polyelectrolytes interacting with growth factors and other transient components of the extracellular matrix [78]. In this case, the enrichment derives from a strong overexpression of the transcripts coding for these enzymes in the hybrids and *A. pegreffii* with respect *to A. simplex s.s.* Reversely, the pathway of alanine aspartate and glutamate metabolism is shown as significantly enriched when the hybrids and A*. simplex s.s.* are compared with *A. pegreffii* but not when they were compared each to other thus suggesting that the *A. pegreffii* parental does not predominate in all expression patterns of hybrids. The remaining pathways (pyruvate metabolism, aminoacyl-tRNA biosynthesis, streptomycin biosynthesis, starch and sucrose metabolism, pentose phosphate pathway, and cysteine and methionine metabolism) were found as differentially enriched only in 1 of the 3 performed comparisons, predominantly when *A. pegreffii* was compared against any of the 2 other taxa (Fig. 3). Results sugest that *A. pegreffii* and the hybrids exhibit for these pathways more differentially expressed transcripts than those that would be expected by chance. In fact, the ratio between differentially expressed and assayed transcripts associated to the enrichment of each pathway is predominantly lower when the hybrids are compared with *A. pegreffii* than when they are compared with A*. simplex s.s.* or when *A. simplex s.s.* is compared with *A. pegreffii.* Finally, it is worth to also discuss the detection of streptomycin biosynthesis pathway, which is associated with the over expression of 7 transcripts encoding for 2 type of enzymes, inositol-3-phosphate synthases (lyases) and dTDP-4-dehydrorhamnose 3,5-epimerases (isomerases) in *A. pegreffii* and *A. simplex s.s.* when compared to the hybrids. The expression of the genes coding for these enzymes might have been induced by the antibiotic treatment applied on L3 specimens before the extraction (see methods) thus apparently revealing some deficiencies in the hybrids with respect to their parents of for this metabolic pathway. However, we should to also take into account that Inositol-3-phosphate synthase also participates in the metabolism of inositol phosphates, mainly to produce membrane receptor playing role in signalling, cell growth, apoptosis, cell migration, endocytosis, and cell differentiation [79], while dTDP-4-dehydrorhamnose 3,5-epimerases are also associated to the nucleotide sugar metabolism and with the biosynthesis of dTDP-rhamnose to produce rhamnose. The biosynthesis of dTDP-rhamnose is a new pathway involved in hypodermal development or in the production of the cuticle or surface coat in larval stages of *C. elegans* and other nematodes [80]. We thus think that inositol-3-phosphate synthases and dTDP-4-dehydrorhamnose 3,5-epimerases of anisakids might link not only to the metabolism of secondary metabolites but also to signalling, nucleotide sugar metabolism and larval development.

**Fig. 4:**
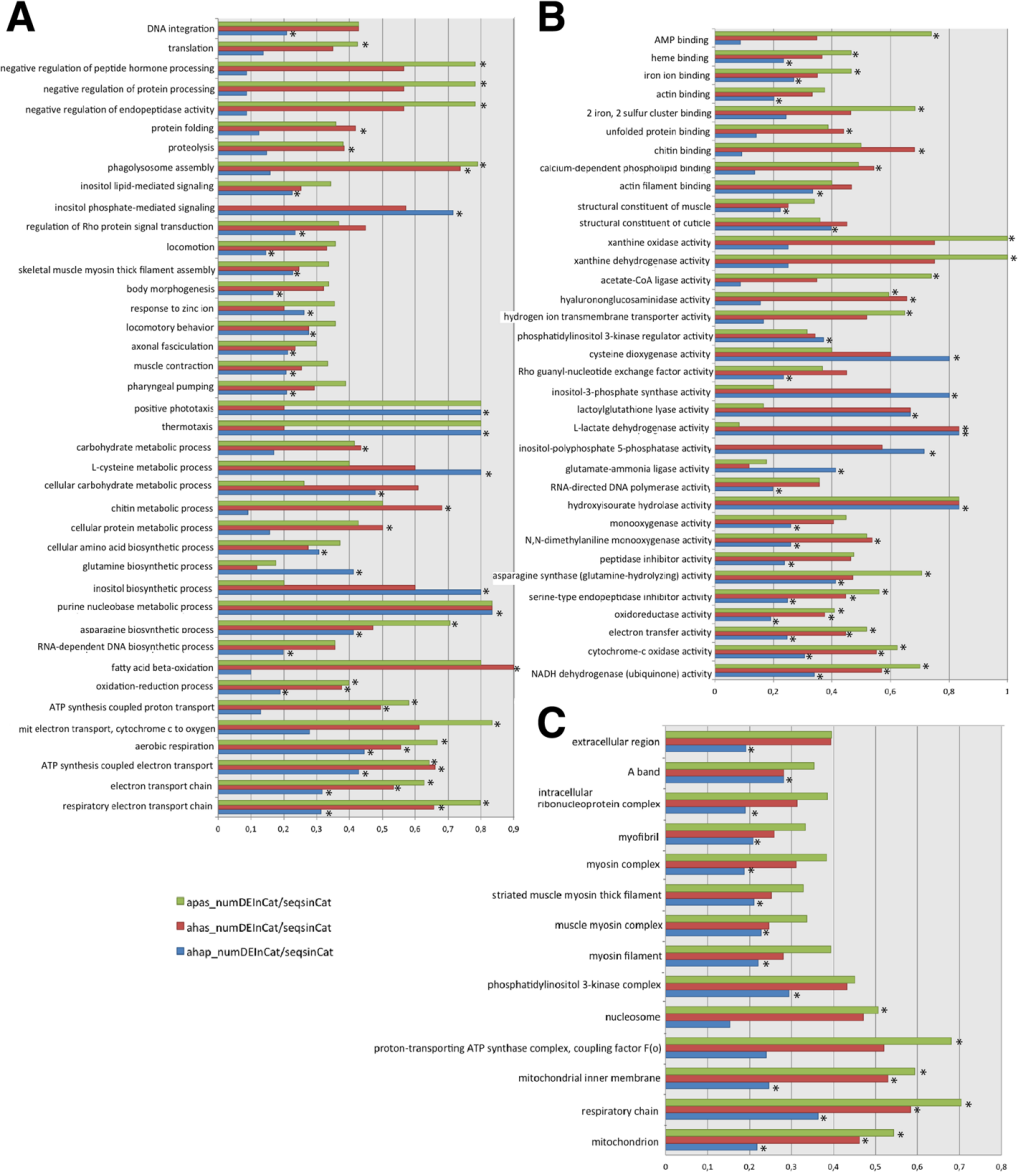
Differential enrichment of Gene Ontologies. **a** Barplot based on the ratio between the number of differentially expressed transcripts and the assayed transcripts with annotation of biological process GOs detected as differentially enriched in at least 1 of the 3 comparisons performed to asses GO enrichment. Plots based on the comparison performed between the hybrids and *A. pegreffii* is colored blue; plots for the comparison between the hybrids and *A. simplex s.s.* are colored red; and those based on the comparison between *A. pegreffii* and *A. simplex s.s.* are colored green. GOs that were significant at FDR < 0.05 are highlighted with an asterisk. **b** Same Bar plot representation based on Molecular Function GOs; **c** Cellular components

Analyses of GO enrichment were also performed based on the 1211 GOs shared by the 3 transcriptomes and revealed differential enrichment of 91 GOs at FDR < 0.05 in at least on of the 3 performed comparisons (Additional file 4). Forty of these 91 correspond to biological processes terms, 37 are molecular functions and 14 cellular components (Fig. 4). Thirteen of these 91 were significant in all comparisons and correspond with functions, processes and sub-localizations associated with the energy metabolism and this mainly includes oxidases, oxidoreductases, dehydrogenases and other enzymes and binding proteins with assigned roles in redox processes, and electron carriers probably related to the transmembrane electrochemical gradient as well as roles for heme-biding proteins (Cytochrome P450s). We think that the latter might also be related with sterol and heme recruitment. As other nematodes, anisakids probably recruit exogenous sterol and heme recruited from host sources to implement sterol and heme dietary needs. Consistent with this argument, the 3 transcriptomes addressed in this study present annotations of prenyltransferases, squalene oxidases and sterol regulatory element-binding proteins (SREBP); prenyltransferases play role in the synthesis of pyrophosphate and geranylpyrophosphate, a major metabolic chokepoint in de novo sterol biosynthesis pathway [81] while squalene and derivatives are the main source of host sterol and SREBP is known to stimulate sterol biosynthesis. The enrichment of the remaining GOs shown in Fig. 4 was significant in 1 or 2 of the 3 performed comparisons and refers to molecular functions biological processes and cellular components related to the enriched pathways shown and discussed above but also with transposition (mainly integrases and proteases encoded by *Bel/Pao* LTR retrotransposons), transport, growth and development, locomotion, and with the extracellular region (ES proteins, allergens and cuticle collagens). In broad terms and for almost all evaluated GOs (with several exceptions) the ratio between differentially expressed and assayed transcripts is again lower if the hybrids are compared with *A. pegreffii* than when the hybrids are compared with A. *simplex* s.s. or when the latter is compared with *A. pegreffii*. In turn, the comparison between the hybrids and *A. pegreffii* presents largely more enrichment than the others; of the 91 GOs shown as differentially enriched, 67 terms are significant in the aforesaid comparison between the hybrids and *A. pegreffii* while 27 GOs are significant when the hybrids and A. *simplex* s.s compared and 34 when the latter is compared with *A. pegreffii*. Interestingly, the most prominent differences in GO enrichment observed between the hybrids and the 2 parents derive from the differential expression of transcripts related with the inositol polyphosphate 5-phosphatase activity involved in inositol phosphate-mediated signaling. While these 2 GO terms are significantly enriched when they are compared with their parental species but no when the parents are compared each to other. Indeed, we think that the deregulation of the inositol polyphosphate 5-phosphatase enzyme and how it affects the whole inositol phosphate-mediated signalling pathway it is worth to be investigated, moreover if we take into account that hybrids are not able to reach adult stages and that inositol phosphate-mediated signalling has been associated to longevity, growth and reproduction in nematodes and other invertebrates [82]. The enrichment of GOs is also consistent with the perspectives provided by the differential expression analyses and the enrichment of metabolic pathways that together supports the idea of that although the alleles of the A. pegreffii parental apparently predominate over those of A. simplex s.s. in the expression patterns of L3 larval stage hybrids the latter are intermediate biological entities with metabolic and functional requirements, quantitatively different to those of the two parental species.

**Fig. 5:**
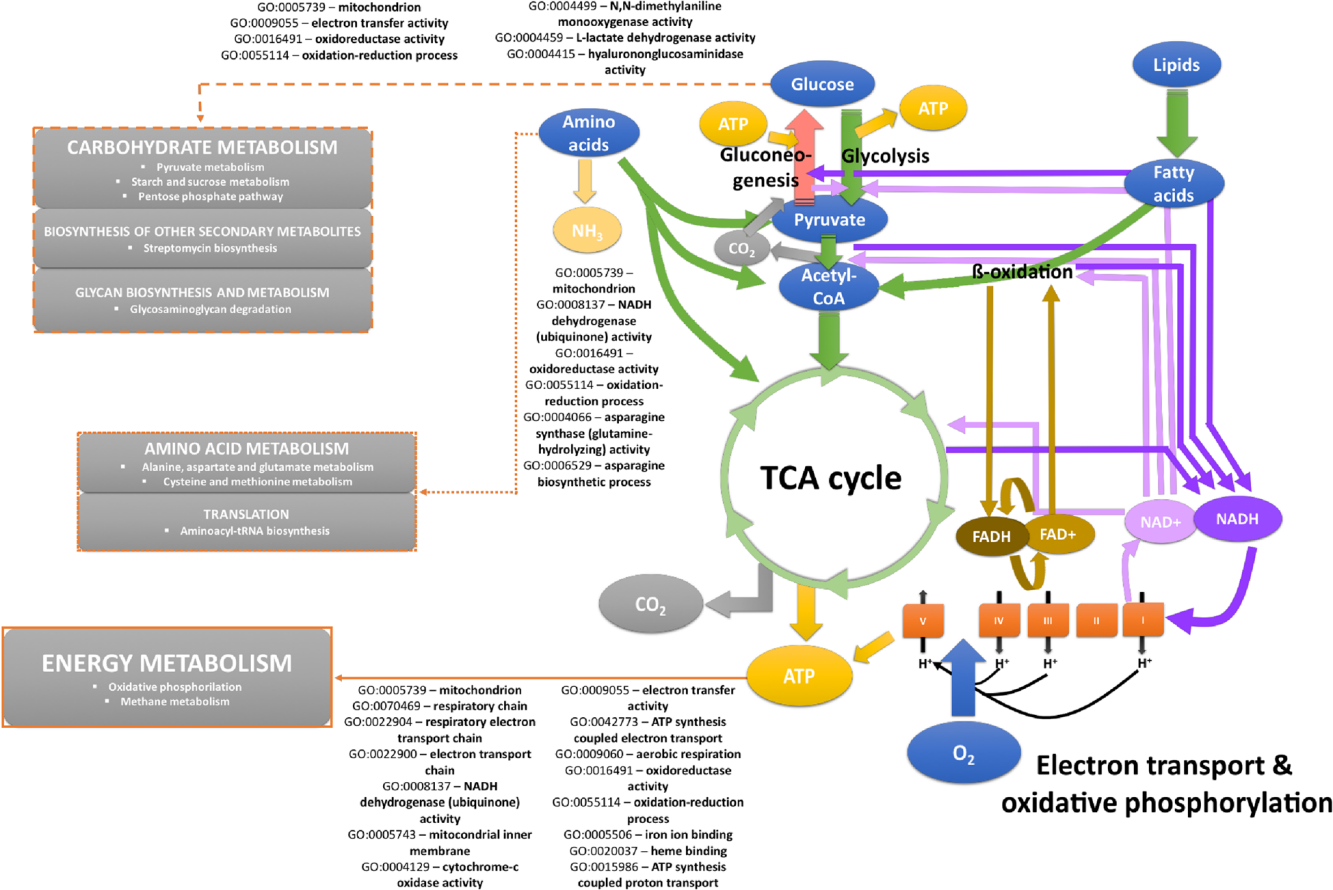
Nematode intermediary metabolism, as described for *C. elegans* and other species. The main pathways and GOs found as enriched among the 3 *Anisakis* transcriptomes are highlighted

The set of metabolic pathways and GOs (i.e. the functional profiles) of *A. simplex s.s., A. pegreffii* and their hybrids are consistent with the aerobic intermediary metabolism of nematodes thus supporting the idea that almost all pathways typically observed in *C. elegans* and other nematodes are present also in these anisakids. A graphical idealisation of the intermediary metabolism of nematodes is represented in Fig. 5 according to [81–85] and the most representative pathways and GOs detected in the 3 *Anisakis* transcriptomes have been highlighted. Our conclusion is that the *A. pegreffii* parental predominates over *A. simplex s.s*. in the expression of hybrids albeit the latter, and *A. pegreffii* also present significant differences between them indicating that hybrids are intermediate biological entities with metabolic and functional requirements quantitatively different to those of their parental. The hybrids thus offer an excellent opportunity to investigate the mechanisms of speciation in *Anisakis* including the novel biological and ecological processes where hybrid genotypes might have a relevant role or significant lacks. Hybridization among *A. simplex s.s*. and *A. pegreffii* has indeed an important role in the evolutionary biology of these 2 species through gene introgression [86]. While hybrids do not appear to reach the definitive host, allowing development to reproductive adults, they reflect the dynamics of moving areas of sympatry. Hybrids may thus be crucial in understanding the microevolutionary processes active in *A. simplex s.l.,* in host-parasite ecology (including the range of paratenic hosts exploited), and in whale migrations (as interbreeding must take place in whales as final host).

### Genes encoding for excretory/secretory proteins

**Fig. 6:**
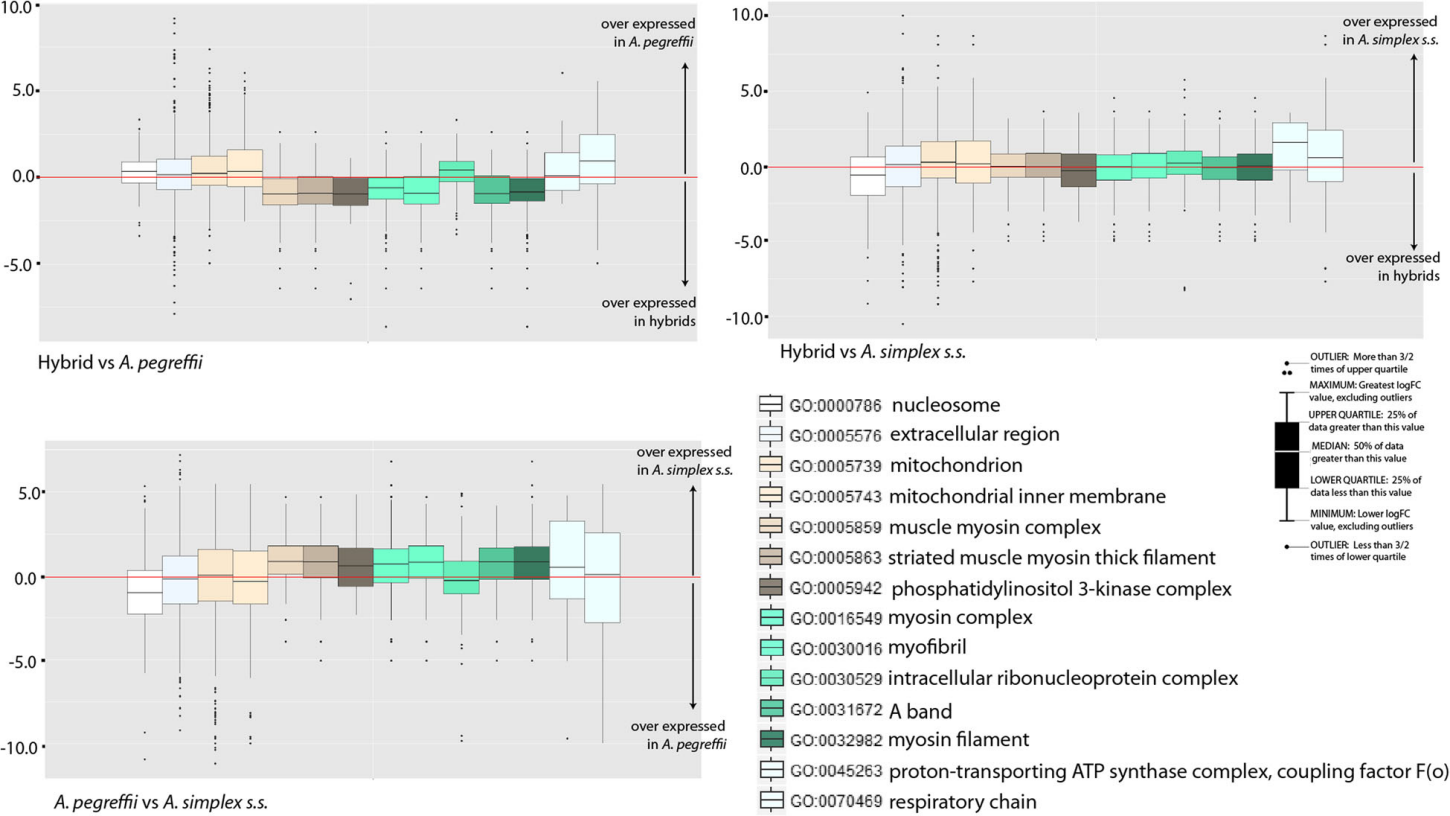
LogFCs for differentially enriched Cellular Component GOs. Box plots based on the median, quartiles, maximum and minimum and outlier values of the LogFC of all transcripts contributing to the enrichment of Cellular Component GOs per differential expression comparison. Red lines highlight the logFC value of zero

**Fig. 7:**
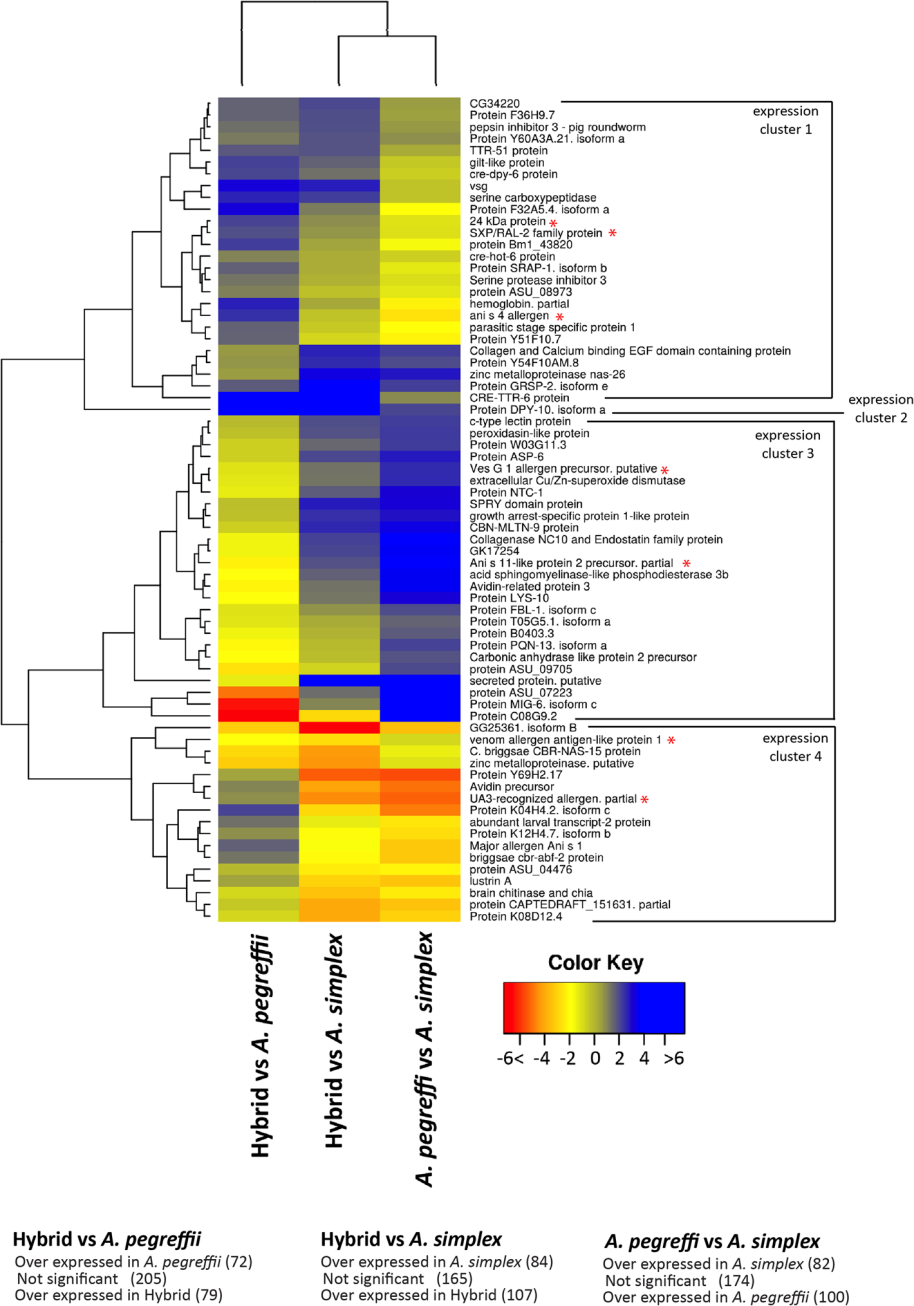
Expression patterns of excretory/secretory genes. Heatmap with double clustering based on the LogFCs of 70 representative ES genes (rows) identified as differentially expressed at a FDR < 0.05 in at least 2 of the 3 differential expression comparisons (columns). Quantiles 0.05, 0.5 and 0.95 were used as breaks to color the transcript counts in red, yellow and blue, respectively. Below is, the color scale used to color the gradient of LogFC values. Columns and rows were clustered using the complete linkage with euclidean distance measure. For a logFC summary of the 356 genes assayed see Additional file 5

To specifically focus on the potential sub-localizations of the products encoded by differentially expressed transcripts associated to enrichment we created a box plot based on the logFCs of transcripts associated to enriched cellular component GOs (Fig. 6). The analysis shows the relationships of over/under expression observed among the 3 taxa with respect to products sub-localized in mitochondrion, respiratory chain, ATP transporting factors and myosin complexes with ATP-dependent roles in muscle contraction, motility and the extracellular region. A significant number of outliers (due to their extremely high logFCs) correspond to transcripts whose product is sub-localized in the extracellular region thus indicating relevant differences with respect to genes encoding likely ES proteins. All ascaridids, *Anisakis* included, release ES products to the host environment. Infective L3 penetrate the intestinal wall of the fish host, and may encyst on the surface of the internal organs or migrate to the muscular tissues. The migration may occur not only when the host is alive [87–91] but also after death [92–94]. These processes are likely to be mediated by secreted enzymes. ES proteins have additional functional roles connected to nutrition, infectivity, allergy, immune evasion or pathogenicity [34, 95, 96] playing important roles in the host-parasite interaction. In the aforeshown analyses performed at the whole-transcriptome level we showed that part of the differential enrichment between taxa corresponds to transcripts encoding likely ES proteins (above). According to our annotations these transcripts correspond to the expression of at least 356 non-redundant gene descriptions of which we create a count file of mapped reads to explore how different are the expression patterns of the investigated taxa with respect to ES genes (see Methods). Nearly three-quarters (266/356) were found to be significantly differentially expressed in one or more comparisons under a FDR < 0.05 as threshold cutoff (Additional file 5). More specifically, 151 genes were found as differentially expressed when comparing hybrids and *A. pegreffii*. Of these, 79 genes were overexpressed in hybrids and 72 overexpressed in *A. pegreffii*. Comparing hybrids with *A. simplex s.s.,* identified 191 differentially expressed genes, of which 107 were overexpressed in hybrids and 84 in *A. simplex s.s..* Comparing *A. simplex s.s.* and *A. pegreffii*, 100 transcripts were overexpressed in *A. pegreffii* and 82 in *A. simplex s.s*. A heatmap with clustering of the logFC per comparison of the 70 most differentially expressed ES genes (Fig. 7) permits to visualize four major patterns of expression. Rows (i.e. the gene expression patterns) split in 4 Clusters of expression designated as clusters 1,2,3 and 4. Cluster 1 groups genes over-expressed in *A. pegreffii* compared to both *A. simplex s.s.* and hybrids, cluster 2 is a single gene, overexpressed in hybrids. Cluster 3 is defined by genes over-expressed in *A. simplex* s.s. compared to both *A. pegreffii* and hybrids, and genes belonging to Cluster 4 were predominantly over-expressed in both A. *pegreffii* and hybrids compared to *A. simplex s.s*. ES genes with allergenic properties are highlighted with red asterisks. In particular, the analysis shows that in mixed infections different species and hybrids of *Anisakis* may produce different kinds and levels of ES proteins, or in other words that the modulation of the host environment may depend on the mix of larvae from species and hybrids. Considering that hybrids are not able to complete their life cycle and that no colony of L3 hybrids has been yet described in nature parasiting alone their hosts (as far as we know hybrids have been only reported accompanied by their parental species) it is tempting to speculate based on our results that hybrids might be able to survive in host environments only when they are accompanied by the parental species. Facilitation may also derive from genes overexpressed or downexpressed in the hybrids.

### Detection and classification of allergens

Anisakiasis is a consequence of allergic reaction to several allergenic molecules expressed and/or secreted by *Anisakis* in their intermediate hosts. Briefly, 15 allergens (Ani s 1 to Ani s 14 and Ani s troponin C) are formally considered from *A. simplex s.s.* according to the AllFam database of allergen families and the WHO/IUIS Allergen Nomenclature Database [13, 14]. Five of the 15 *Anisakis* formal allergens - Ani s1, and Ani s 2 and Ani s 3, Ani s 7, and Ani s 12 - are considered to be major allergens (one to which > 50% of allergic patients immunologically react and present unusual levels of IgE and IgG in serum [97, 98]); Ani s 1 belongs to the animal Kunitz serine protease inhibitor family of protease inhibitors (AF003); Ani s 2 is a myosin heavy chain family (AF100) [99] that includes the highly cross-reactive mite paramyosin major allergens [100]; Ani s 3 is part of the tropomyosin family (AF054), which have been identified as minor inhalative allergens in arthropods and as major food allergens in crustaceans, mollusks and *Anisakis* [101]; and Ani s 7 and Ani s 12 are 2 proteins with unknown biological functions. The remaining *Anisakis* allergens are minor allergens; Ani s 4 belongs to the cystatjn family (AF005) [102] of reversibly binding cysteine protease inhibitors; Ani s 6 belongs to the cysteine-rich trypsin inhibitor-like domain family [103] (AF027); Ani s 5, Ani s 8 and Ani s 9 are members of the nematode-specific SXP/RAL-2 family (AF137), which have unknown function [104, 105]; Anis s 10, Ani s 11 and Ani s 14 similarly remain functionally unclassified [106–108]; Anis s troponin C is a member of the EF hand family (AF007), which has roles in calcium signaling and binding [109]; and Ani s 13 belongs to the globin family (AF009) [110]. Yet more interesting, recent proteomic analyses have identified up to 28 potential new allergens in *Anisakis* that can be classified into distinct allergen families [15] and a more recent transcriptomics study [16] has identified several new allergens from *A. simplex s.s.* and *A. pegreffii.* Additional potential allergens (Ani s 24 kDa, Ani s CCOS3, Ani s cytochrome B, Ani s FBPP, Ani s NADHDS4L, Ani s NARaS and Ani s PEPB) have been also considered according to previous studies [111–113]. All these molecules are classified or can be classified as food allergens because they are present in marine fish. The characterization of allergens in anisakids is still open and that will aid diagnosis and possible alleviation of the serious symptoms of anisakiasis. A complete allergome (all allergen proteins) catalogue for anisakids is required.

Special importance should be paid to proteins related to mechanisms of parasitism and pathogeny of these nematodes. According to a recent transcriptomic study [114] in the pharingeal region of *A. simplex s.s.* there are at least 226 transcripts which are potentially involved in invasion and host pathogen interplay. The same study found at least 339 transcripts with a similar role in *A. pegreffii*. Regarding this, 13 immunoreactive proteins from the esophageal glands of *A. simplex s.s.* have been described as potential allergens (data not published; PARASITE project [115].

We created a protein sequence database including 509 non-redundant food allergens from fungi, plants and animals (see Methods). This database was used to search homologues in the *Anisakis* consensus transcriptome we had assembled in this study. We detected high-scoring matches (E-values < 1.99E-06) to 937 consensus transcripts from 121 different allergens that can be classified into 74 families of allergens following the criteria of classification contemplated by AllFamDB and WHO/IUIS 13,14] and other classificatory criteria [111–113] (Table 3; Additional file 6). Of the 74 identified families, 41 were detected through similarity to allergens described from animals (15 correspond to all *Anisakis* formal allergens), 15 were detected using fungal allergens and 13 using allergens of plants (i.e. some of these had best matches with allergens from fungi and plants). The remaining 5 sequences were identified using allergens present in more than one realm of life. Differential expression analyses revealed the 74 families to be present in the 3 transcriptomes (Additional file 6). Thirty one families were found as differentially expressed (under a FDR threshold of 0.05) when comparing hybrids to *A. pegreffii*, 33 families when comparing hybrids to *A. simplex s.s.,* and 36 families when comparing *A. pegreffii* to *A. simplex s.s*. These expression patterns are consistent although more slightly with the other analyses performed by us (the *A. pegreffii* parental genotype slightly predominates over *A. simplex s.s* in the hybrids’ expression patterns). It is striking that all 3 taxa express a large number of different allergen types, many more than has been described previously. So the potential allergen repertoire of these anisakids can be expanded. We think that this repertory might be related to (or be able to partially explain) the cross-reaction of anisakiasis patients to allergens from other sources. It is usually assumed that after sensitization by nematode L3, subsequent contact with these same allergens is required to induce an allergenic response [116]. A number of *Anisakis* allergens are ES proteins [15], with high affinity to specific immunoglobulin E (IgE) [117], and patients primed by *A. simplex s.s.* might respond adversely to cross-reactive antigens of not only other anisakids but also of other nematodes and arthropods [118]. Somatic allergens such as Ani s 3 (tropomyosin) cross-react with proteins of crustaceans, molluscs and insects (particularly the American cockroach) [119].

**Table 3:** Distribution of Allergen Families (AllFams) and logFC results from differential expression

Allergen Families	FamID	LogFCs Hh vs *Ap*	LogFCs Hh vs *As*	LogFCs *Ap* vs *As*	Con Seqs	Allergen Name	Realm	Type of allergen
24 kDa protein	Not yet assigned	**2,24,615**	**0,94,729**	−**1,29,887**	20	NA	Animal	NA
60S acidic ribosomal protein	AF070	**0,70,555**	0,25,574	− 0,44,982	4	Asp f 8, Alt a 12, Asp f 8, Fus c 1	Fungi	Minor
Aldehyde dehydrogenase	AF040	0,06989	−0,10,196	− 0,17,186	26	Blo t 4	Animal	Minor
Alpha-amylase	AF033	−0,05472	0,32,169	0,37,641	6	Alt a 10, Cla h 10	Fungi	Minor
Ani s 10 allergen	Not yet assigned	**1,07632**	0,06603	−**1,01029**	1	Ani s 10	Animal	NA
Ani s 11 allergen	Not yet assigned	−**2,67,162**	**2,26,799**	**4,93,961**	6	Ani s 11, Ani s 11 L1, Ani s 11 L2	Animal	NA
Ani s 12 allergen	Not yet assigned	−0,22,028	−0,25,132	− 0,03103	4	Ani s 12	Animal	Major
Animal Kunitz serine protease inhihibitor	AF003	**1,50,377**	**−1,17,032**	**−2,67,407**	9	Ani s 1	Animal	Major
ARM-like	Not yet assigned	**0,88,344**	**−4,73,273**	**−5,61,615**	44	Ani s 7, Ani s 14	Animal	Major, NA
ATP:guanido phosphotransferase	AF049	0,28,397	0,03604	−0,24,793	5	Pen m 2, Der p 20, Plo i 1	Animal	Major, Minor
ATP synthase	Not yet assigned	0,41,629	0,19,251	−0,22,378	3	Bos d OSCP	Animal	Minor
BCL7 family	AF121	−0,42,399	0,29,518	0,71,916	1	Hom s 3	Animal	NA
Calreticulin family	AF055	−0,27,705	**− 0,65,631**	− 0,37,927	7	Pen ch 31	Animal	NA
CAP family	AF044	**− 0,63,714**	**−1,22,008**	**−0,58,293**	12	sol i 3, Dol a 5, Vesp c5, Poly s 5, Pol d 5, Dol m 5, allrgn_V5/Tpx1	Animal	Major, NA
Carboxylesterase	AF140	−0,07113	−0,09074	− 0,01961	50	Api m 8	Animal	Minor
Casein kinase II regulatory subunit	Not yet assigned	−0,54,905	**−0,81,674**	− 0,26,768	2	Gal d Phosvitin	Animal	Na
Catalase	AF047	**1,53,912**	**0,81,466**	**−0,72,447**	1	Pen c 30	Fungi	Minor
Chitinase class III and peritrophin-like	AF077	−0,55,642	−0,22,104	0,33,539	12	Der p 15, Der f 15	Animal	Major
Collagen	AF097	−0,20,422	0,10,867	0,31,289	2	Bos d alpha 2 l	Animal	NA
Cyclophilin	AF038	0,18,030	0,27,158	0,09128	13	Asp f 27	Fungi	NA
Cystatin	AF005	**2,62,999**	−0,32,427	**−2,95,425**	4	Ani s 4	Animal	NA
Cysteine-rich trypsin inhibitor-like domain	AF027	**−2,05519**	**0,03836**	**2,09355**	5	Ani s 6	Animal	NA
Cytochrome b-c1	Not yet assigned	**0,59,193**	0,94,815	0,35,621	6	NA	Animal	NA
Cytochrome B5	Not yet assigned	1**,95,134**	0,37,530	**−1,57,620**	3	NA	Animal	NA
Cytochrome c	AF006	0,14,607	**1,04446**	**0,89,838**	5	Cur l 3	Fungi	Major
EF hand family	AF007	0,20,495	**0,40,417**	0,19,922	34	Bla g 6, Per a 6, Lit v 3, Ani s Troponin C	Animal	Major, Minor, NA
Enolase	AF031	**−0,52,169**	−0,22,686	0,29,484	5	rho m 1, hev b 9, Alt a 6	Fungi,Plant	Minor
Eukaryotic aspartyl protease	AF004	−0,11,780	−0,35,758	− 0,23,979	14	Bla g 2	Animal	Major
Eukaryotic elongation factor 1	AF011	0,03983	−0,05788	−0,09771	8	Pen c 24	Fungi	NA
Fe/Mn superoxide dismutase	AF020	0,00817	0,19,412	0,18,595	2	Hev b 10	Plant	Minor
Gelsolin family	AF074	**−0,81,825**	**−0,61,973**	**0,19,852**	17	Der f 16	Animal	Minor
GILT family	AF155	**1,61,144**	**0,92,869**	−0,68,276	3	Tri a 27	Plant	NA
Globin	AF009	**2,89,321**	**0,55,871**	**−2,33,450**	3	Ani s 13	Animal	Minor
Glutathione S-transferase	AF010	0,06592	0,47,962	0,41,370	11	Bla g 5, Der p 8	Animal	Major
Glyoxalase superfamily	AF082	−0,40,931	0,77,379	**1,18,310**	2	Ory Glyosalase l	Plant	NA
GMC oxidoreductase	AF081	0,37,676	0,45,602	0,07926	10	Mala s 12	Fungi	Major
Heat shock protein Hsp70	AF002	−0,17,513	**−0,64,037**	−**0,46,524**	45	Pen c 10, Cla h HSP70	Fungi	Minor
Heat shock protein Hsp90	AF042	−0.19335	0.11856	**0.31191**	15	Asp f 12	Fungi	NA
Hevein-like domain	AF043	0.35419	−0.39541	**−0.74952**	1	Hev b 11	Plant	Minor
Histidine acid phosphatase	AF062	−0.03153	**−0.32299**	**− 0.29146**	15	Api m 3	Animal	Minor
Hyaluronidase	AF103	**1.02016**	−0.32364	−**1.34380**	10	Pol a 2, Api m 2	Animal	NA. Major
Intermediate filament protein	AF008	**−0.30492**	**−0.89969**	**− 0.59477**	13	Hom s 5	Animal	NA
Lactate/malate dehydrogenase	AF014	**−0.78551**	**−0.56985**	0.21566	3	Cit la MDH	Plant	Major
Larval allergen (Brugia malayii)	Unclassified	0.32808	**1.48199**	**1.15313**	2	NA	Animal	NA
Lipase	AF037	0.09487	**−0.64847**	**−0.74333**	15	Dol m1, Pol d 1, rhi o Lipase, The l lipase	Animal. Plant	Major. NA
Lipocalin	AF015	**0.51183**	0.05554	**−0.45629**	3	tyr p 13	Animal	Minor
Major allergen (Loa loa)	Unclassified	−0.00868	−0.22329	− 0.21461	4	NA	Animal	NA
Myosin heavy chain	AF100	**−0.21310**	0.07600	**0.28910**	179	Ani s 2, Blo t 11, Der f 11, der p 11	Animal	Major. Minor
NAC domain	AF107	**0.58475**	0.39473	−0.19002	3	Hom s 2	Animal	NA
Nitrophorin	Unclassified	0.27507	0.31344	0.03836	5	Cim I Nitrophorin	Animal	NA
Papain-like cysteine protease	AF030	−0.01475	0.08466	0.09941	24	Act d 1, Ana c 2, car p 1, Gly m Bd 30 k, blo t 2	Animal.Plant	Major. NA
Patatin family	AF104	−0.27609	**−3.25600**	**−2.97961**	1	Hev b 7	Plant	Major
Phosphoglycerate kinase	Unclassified	−0.22025	**−0.55632**	− 0.33607	5	Can a PGK	Fungi	NA
Profilin family	AF051	0.19390	−0.08435	−0.27824	1	Par j 3	Plant	Major
Prolamin superfamily	AF050	0.07050	−0.07100	−0.14148	1	Tri a 26	Plant	Major
Prolyl oligopeptidase family	AF061	−0.04633	0.30623	0.35255	6	Ves v 3, Tri r 4	Animal, Fungi	Major. NA
Proteasome subunit beta type	Unclassified	0**.98838**	**1.02852**	0.04012	3	Zea m 20S	Plant	Minor
Protein kinase	AF017	**−0.43020**	−0.00592	**0.42428**	101	Sal k 2	Plant	Major
Ribosomal protein L3	AF058	**1.01034**	**1.22633**	0.21599	2	Asp f 23	Fungi	Minor
SART-1 family	AF116	−0.01191	−0.21989	−0.20797	1	Hom s 1	Animal	NA
Serpin serine protease inhibitor	AF018	**0.84445**	0.38305	**−0.46140**	12	Gal d 2	Animal	Major
Short-chain dehydrogenase	AF028	−0.09468	0.02563	0.12031	19	Alt a 8, Cla h 8	Fungi	Major. Minor
Subtilisin-like serine protease	AF021	0.06703	**−1.01313**	**−1.08002**	1	Rho m 2	Fungi	Major
SXP/RAL-2 family	AF137	**1.79413**	**0.74628**	**−1.04786**	15	Ani s 8, Ani s 9, Ani s 5	Animal	Minor
Thioredoxin	AF023	0.57209	**0.74939**	0.17728	1	Asp f 28	Fungi	Major
Translationally controlled tumour protein	AF136	0.40520	−0.40064	**−0.80584**	3	Hom s TCTP	Animal	NA
Triosephosphate isomerase	AF032	0.01199	0.03933	0.02734	2	Tri a 31	Plant	Minor
Tropomyosin	AF054	**−0.31997**	**−0.39583**	−0.07586	17	Ani s 3, Bal r 1, Pat y 1, Hal d 1, Hel as 1, Por s 1, Der p 10, Pan s 1, Cra g 1	Animal	Major. Minor. NA
Troponin I and T	AF146	0.23760	− 0.36364	**− 0.60124**	2	Pon l 7	Animal	Minor
Trypsin-like serine protease	AF024	−0.33264	0.37800	**0.71063**	4	Blo t 3, Api m 7, Der f 6	Animal	Minor. NA
Tuber Storage Protein	Unclassified	0.26964	**0.69673**	**0.42708**	5	Dio o TSP	Plant	NA
Tubulin/FtsZ family	AF025	−0.22307	**−0.26109**	−0.03802	46	Lep d alpha Tubulin, Tyr p alpha Tubulin	Animal	Minor
WD-40 repeat	AF142	0.00385	−0.15642	−0.16027	15	For t 2	Plant	Major
Zn-containing dehydrogenase	AF029	0.02937	−0.01838	−0.04775	5	can a 1	Fungi	Major

List from A to Z, Significant differential expressed AllFams supported by FDR < 0,05 are higlighted bold, NA means not avaiable information

## Conclusions

*A. simplex s.s*., *A. pegreffi* and their hybrids show differences in their gene expression patterns in the larval L3 stage. Strong parent-of-origin effects were observed indicating that hybrids are intermediate biological entities among their parental species, and thus of great interest in the study of speciation in nematodes. Differential expression analyses based on genes coding for secreted proteins suggests that co-infections present different repertoires of released proteins to the host environment. Both species and their hybrids, share more allergen genes than previously thought and are likely to induce overlapping disease responses. In the protein sequence database which includes 509 non-redundant food allergens from fungi, plants and animals we detected 121 different allergens belonging to 74 allergen families following recognized classificatory criteria.

## Additional files

Additional file 1: Phylogenetic relationships of the considered populations as an individual’s selection basis for RNA sequencing of *A. simplex s.s*., *A. pegreffii* and their hybrid haplotype. Tree was obtained based on maximum likelihood (ML) and Bayesian inference analysis using mitochondrial COII gene and the GTR + I + G evolutionary model. Numbers at nodes correspond to ML bootstrap proportions (BP) (above number) and Bayesian posterior probabilities (BPP) (under number). (PNG 621 kb)

Additional file 2: List of species and specimen used in the phylogenetic tree of Additional file 1**.** Code of the voucher specimen and accession number for mitochondrial gene COII (*: sequences obtained from GenBank). Labeled are the specimens selected for RNA sequencing (first number, population; second number specimen)*. A. simplex s.s. – A. pegreffii* refers to hybrids haplotype according Abollo et al. [23]. (DOCX 47 kb)

Additional file 3: Differential expression analyses at the whole transcriptome level. Results obtained from the analyses of differential expression; 1) Hybrids vs *A. pegreffi*; 2) Hybrids vs *A. simplex s.s*.; 3) *A. pegreff*ii vs *A. simplex s.s*. Rows summarizing significant differentially expressed transcripts supported by FDR < 0.05) are highlighted in light blue. (XLSX 12115 kb)

Additional file 4: Differentially enriched metabolic pathways and GOs. Annotation of metabolic pathways and and GOs integrating results of differential enrichment using GOseq. (XLSX 232 kb)

Additional file 5: Differential expression of ES transcripts. 1) Count file used as input in the differential expression test performed at the ES gene level; 2) Summarization of the 356 ES genes accompanied by logFC values and their FDR support obtained from the 3 performed differential expression analyses and additional information relative to consensus sequences grouped to each gene description, as well as associated GOs terms and identification of SignalP domain. This summarization is also available in AnisakisDB as a dynamic table from where sequences and annotations can be navigated and retrieved. (XLSX 91 kb)

Additional file 6: *Anisakis* allergome. 1) Count file and results of differential expression for the 74 allergen families detected in *A. simplex s.s*., *A. pegreffi* and hybrids. 2) Blast results obtained in the search performed using 509 known allergens as queries against the reference *Anisakis* transcriptome. Nine hundred and thirty seven consensus sequences of the reference transcriptome were detected to significantly match to the queries and were classified into 74 allergen families of which we include additional information. This summarization is also available in AnisakisDB as a dynamic table from where sequences and annotations can be easily navigated and retrieved. (XLS 351 kb)

## Abbreviations

AllFam: Allergen family
BCV: Biological coefficient of variation
BIC: Bayesian information criterion
BPP: Bayesian posterior probability
COII: Cytochrome oxidase subunit II
CPM: Counts per million
EC: Enzyme commission
ENA: European Nucleotide Archive
ES: Excretory/secretory
FDR: False discovery rate
GI: Entrez Gene Identifier
GO: Gene Ontology
hybrid: Hybrid haplotype
IgE: Immunoglobulin E
ITS: Internal transcribed spacers
KOG: Eukaryotic orthologous groups
L3: Third-stage larvae
logFC: Log2 fold change
MDS: Multidimensional scaling
ML: Maximum likelihood
NR: NCBI non-redundant protein database
NT: NCBI nucleotide database
ORF: Open reading frame
TM: Transmembrane domain
