# Supplementary figures and images for: Functional insights into the infective larval stage of Anisakis simplex s.s., Anisakis pegreffii and their hybrids based on gene expression patterns
Source: BMC Genomics. 2018 Aug 7;19:592. doi: 10.1186/s12864-018-4970-9 (PMC6080401; doi:10.1186/s12864-018-4970-9)

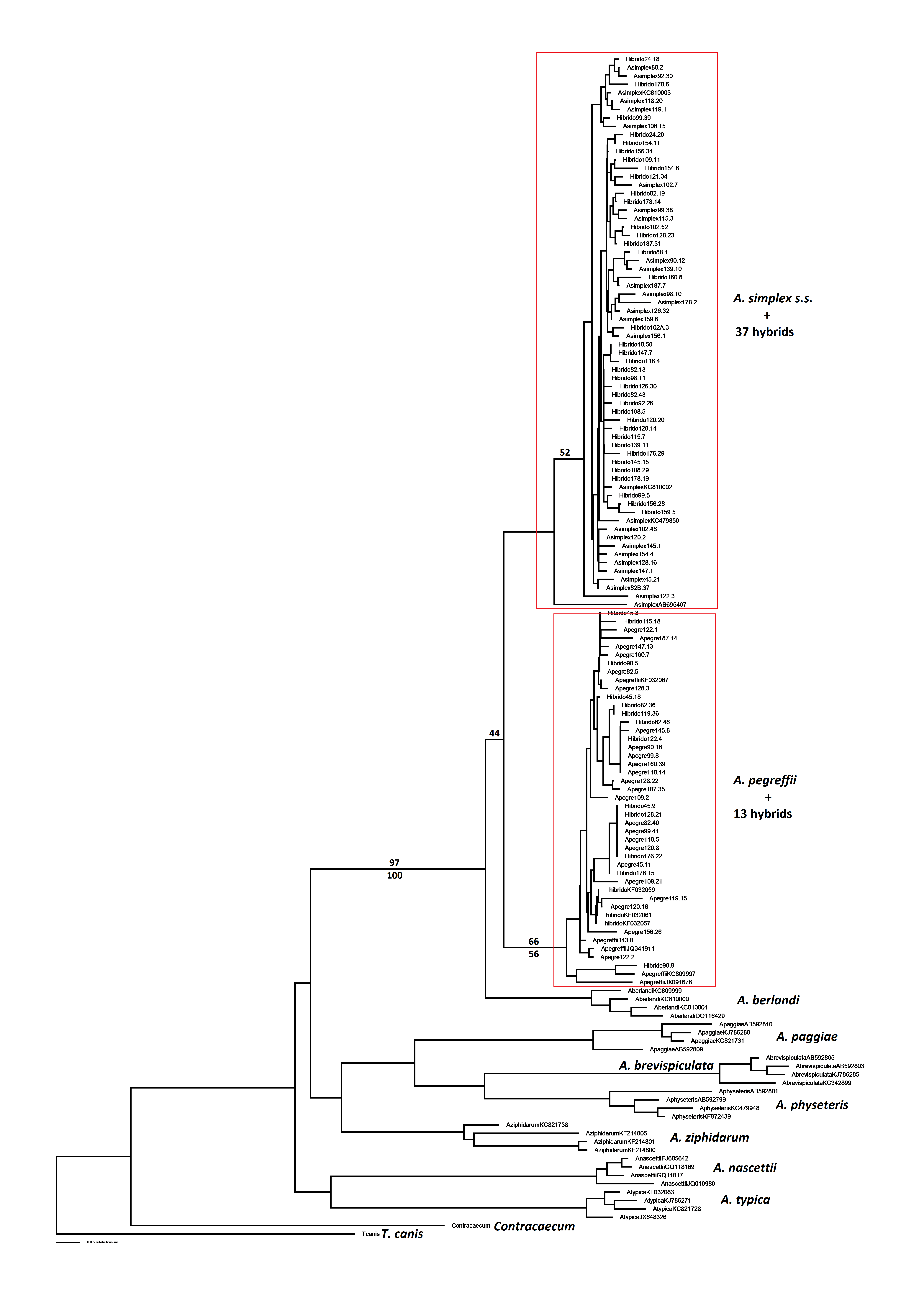

Supplement: Supplementary file 1 — Phylogenetic relationships of the considered populations as an individual’s selection basis for RNA sequencing of A. simplex s.s., A. pegreffii and their hybrid haplotype. Tree was obtained based on maximum likelihood (ML) and Bayesian inference analysis using mitochondrial COII gene and the GTR + I + G evolutionary model. Numbers at nodes correspond to ML bootstrap proportions (BP) (above number) and Bayesian posterior probabilities (BPP) (under number). (PNG 621 kb) [file 12864_2018_4970_MOESM1_ESM.png]
