# Supplementary material for: Functional insights into the infective larval stage of Anisakis simplex s.s., Anisakis pegreffii and their hybrids based on gene expression patterns
Source: BMC Genomics. 2018 Aug 7;19:592. doi: 10.1186/s12864-018-4970-9 (PMC6080401; doi:10.1186/s12864-018-4970-9)
Supplement: Supplementary file 2 — List of species and specimen used in the phylogenetic tree of Additional file 1. Code of the voucher specimen and accession number for mitochondrial gene COII (*: sequences obtained from GenBank). Labeled are the specimens selected for RNA sequencing (first number, population; second number specimen). A. simplex s.s. – A. pegreffii refers to hybrids haplotype according Abollo et al. [23]. (DOCX 47 kb) [file 12864_2018_4970_MOESM2_ESM.docx]

| Species | Voucher number | EMBL Accession number  COII |
| --- | --- | --- |
| *Anisakis simplex* | 45-21 | LT883269 |
| *Anisakis simplex* | 82B-37 | LT883270 |
| *Anisakis simplex* | 88-2 | LT883271 |
| *Anisakis simplex* | 90-12 | LT883272 |
| *Anisakis simplex* | 92-30 | LT883273 |
| *Anisakis simplex* | 98-10 | LT883274 |
| *Anisakis simplex* | 99-38 | LT883275 |
| *Anisakis simplex* | 102-7 | LT883276 |
| *Anisakis simplex* | 102-48 | LT883277 |
| *Anisakis simplex* | 108-15 | LT883278 |
| *Anisakis simplex* | 115-3 | LT883279 |
| *Anisakis simplex* | 118-20 | LT883280 |
| *Anisakis simplex* | 119-1 | LT883281 |
| *Anisakis simplex* | 120-2 | LT883282 |
| *Anisakis simplex* | 122-3 | LT883283 |
| *Anisakis simplex* | 126-32 | LT883284 |
| *Anisakis simplex* | 128-16 | LT883285 |
| *Anisakis simplex* | 139-10 | LT883286 |
| *Anisakis simplex* | 145-1 | LT883287 |
| *Anisakis simplex* | 147-1 | LT883288 |
| *Anisakis simplex* | 154-4 | LT883289 |
| *Anisakis simplex* | 156-1 | LT883290 |
| *Anisakis simplex* | 159-6 | LT883291 |
| *Anisakis simplex* | 178-2 | LT883292 |
| *Anisakis simplex* | 187-7 | LT883293 |
| *Anisakis pegreffii* | 45-11 | LT883294 |
| *Anisakis pegreffii* | 82-5 | LT883295 |
| *Anisakis pegreffii* | 82-40 | LT883296 |
| *Anisakis pegreffii* | 90-16 | LT883297 |
| *Anisakis pegreffii* | 99-8 | LT883298 |
| *Anisakis pegreffii* | 99-41 | LT883299 |
| *Anisakis pegreffii* | 109-2 | LT883300 |
| *Anisakis pegreffii* | 109-21 | LT883301 |
| *Anisakis pegreffii* | 118-5 | LT883302 |
| *Anisakis pegreffii* | 118-14 | LT883303 |
| *Anisakis pegreffii* | 119-15 | LT883304 |
| *Anisakis pegreffii* | 120-8 | LT883305 |
| *Anisakis pegreffii* | 120-18 | LT883306 |
| *Anisakis pegreffii* | 122-1 | LT883307 |
| *Anisakis pegreffii* | 122-2 | LT883308 |
| *Anisakis pegreffii* | 128-3 | LT883309 |
| *Anisakis pegreffii* | 128-22 | LT883310 |
| *Anisakis pegreffii* | 143-8 | LT883311 |
| *Anisakis pegreffii* | 145-8 | LT883312 |
| *Anisakis pegreffii* | 147-13 | LT883313 |
| *Anisakis pegreffii* | 156-26 | LT883314 |
| *Anisakis pegreffii* | 160-7 | LT883315 |
| *Anisakis pegreffii* | 160-39 | LT883316 |
| *Anisakis pegreffii* | 187-14 | LT883317 |
| *Anisakis pegreffii* | 187-35 | LT883318 |
| *Anisakis simplex* - *Anisakis pegreffii* | 24-18 | LT883319 |
| *Anisakis simplex* - *Anisakis pegreffii* | 24-20 | LT883320 |
| *Anisakis simplex* - *Anisakis pegreffii* | 45-8 | LT883321 |
| *Anisakis simplex* - *Anisakis pegreffii* | 45-9 | LT883322 |
| *Anisakis simplex* - *Anisakis pegreffii* | 45-18 | LT883323 |
| *Anisakis simplex* - *Anisakis pegreffii* | 48-50 | LT883324 |
| *Anisakis simplex* - *Anisakis pegreffii* | 82-13 | LT883325 |
| *Anisakis simplex* - *Anisakis pegreffii* | 82-19 | LT883326 |
| *Anisakis simplex* - *Anisakis pegreffii* | 82-36 | LT883327 |
| *Anisakis simplex* - *Anisakis pegreffii* | 82-43 | LT883328 |
| *Anisakis simplex* - *Anisakis pegreffii* | 82-46 | LT883329 |
| *Anisakis simplex* - *Anisakis pegreffii* | 88-1 | LT883330 |
| *Anisakis simplex* - *Anisakis pegreffii* | 90-5 | LT883331 |
| *Anisakis simplex* - *Anisakis pegreffii* | 90-9 | LT883332 |
| *Anisakis simplex* - *Anisakis pegreffii* | 92-26 | LT883333 |
| *Anisakis simplex* - *Anisakis pegreffii* | 98-11 | LT883334 |
| *Anisakis simplex* - *Anisakis pegreffii* | 99-5 | LT883335 |
| *Anisakis simplex* - *Anisakis pegreffii* | 99-39 | LT883336 |
| *Anisakis simplex* - *Anisakis pegreffii* | 102-3 | LT883337 |
| *Anisakis simplex* - *Anisakis pegreffii* | 102-52 | LT883338 |
| *Anisakis simplex* - *Anisakis pegreffii* | 108-5 | LT883339 |
| *Anisakis simplex* - *Anisakis pegreffii* | 108-29 | LT883340 |
| *Anisakis simplex* - *Anisakis pegreffii* | 109-11 | LT883341 |
| *Anisakis simplex* - *Anisakis pegreffii* | 115-7 | LT883342 |
| *Anisakis simplex* - *Anisakis pegreffii* | 115-18 | LT883343 |
| *Anisakis simplex* - *Anisakis pegreffii* | 118-4 | LT883344 |
| *Anisakis simplex* - *Anisakis pegreffii* | 119-36 | LT883345 |
| *Anisakis simplex* - *Anisakis pegreffii* | 120-20 | LT883346 |
| *Anisakis simplex* - *Anisakis pegreffii* | 121-34 | LT883347 |
| *Anisakis simplex* - *Anisakis pegreffii* | 122-4 | LT883348 |
| *Anisakis simplex* - *Anisakis pegreffii* | 126-30 | LT883349 |
| *Anisakis simplex* - *Anisakis pegreffii* | 128-14 | LT883350 |
| *Anisakis simplex* - *Anisakis pegreffii* | 128-21 | LT883351 |
| *Anisakis simplex* - *Anisakis pegreffii* | 128-23 | LT883352 |
| *Anisakis simplex* - *Anisakis pegreffii* | 139-11 | LT883353 |
| *Anisakis simplex* - *Anisakis pegreffii* | 145-15 | LT883354 |
| *Anisakis simplex* - *Anisakis pegreffii* | 147-7 | LT883355 |
| *Anisakis simplex* - *Anisakis pegreffii* | 154-6 | LT883356 |
| *Anisakis simplex* - *Anisakis pegreffii* | 154-11 | LT883357 |
| *Anisakis simplex* - *Anisakis pegreffii* | 156-28 | LT883358 |
| *Anisakis simplex* - *Anisakis pegreffii* | 156-34 | LT883359 |
| *Anisakis simplex* - *Anisakis pegreffii* | 159-5 | LT883360 |
| *Anisakis simplex* - *Anisakis pegreffii* | 160-8 | LT883361 |
| *Anisakis simplex* - *Anisakis pegreffii* | 176-15 | LT883362 |
| *Anisakis simplex* - *Anisakis pegreffii* | 176-22 | LT883363 |
| *Anisakis simplex* - *Anisakis pegreffii* | 176-29 | LT883364 |
| *Anisakis simplex* - *Anisakis pegreffii* | 178-6 | LT883365 |
| *Anisakis simplex* - *Anisakis pegreffii* | 178-14 | LT883366 |
| *Anisakis simplex* - *Anisakis pegreffii* | 178-19 | LT883367 |
| *Anisakis simplex* - *Anisakis pegreffii* | 187-31 | LT883368 |
|  |  |  |
| *Anisakis simplex** |  | AB695407 |
| *Anisakis simplex** |  | KC479850 |
| *Anisakis simplex** |  | KC810002 |
| *Anisakis simplex** |  | KC810003 |
| *Anisakis pegreffii** |  | JQ341911 |
| *Anisakis pegreffii** |  | JX091676 |
| *Anisakis pegreffii** |  | KC809997 |
| *Anisakis pegreffii** |  | KF032067 |
| *Anisakis simplex* x *Anisakis pegreffii** |  | KF032057 |
| *Anisakis simplex* x *Anisakis pegreffii** |  | KF032059 |
| *Anisakis simplex* x *Anisakis pegreffii** |  | KF032061 |
| *Anisakis berlandi** |  | DQ116429 |
| *Anisakis berlandi** |  | KC809999 |
| *Anisakis berlandi** |  | KC810000 |
| *Anisakis berlandi** |  | KC810001 |
| *Anisakis nascettii** |  | FJ685642 |
| *Anisakis nascettii** |  | GQ118169 |
| *Anisakis nascettii** |  | GQ118171 |
| *Anisakis nascettii** |  | JQ010980 |
| *Anisakis typica** |  | JX648326 |
| *Anisakis typica** |  | KC821728 |
| *Anisakis typica** |  | KF032063 |
| *Anisakis typica** |  | KJ786271 |
| *Anisakis paggiae** |  | AB592810 |
| *Anisakis paggiae** |  | AB592809 |
| *Anisakis paggiae** |  | KC821731 |
| *Anisakis paggiae** |  | KJ786280 |
| *Anisakis physeteris** |  | AB592799 |
| *Anisakis physeteris** |  | AB592801 |
| *Anisakis physeteris** |  | KC479948 |
| *Anisakis physeteris** |  | KF972439 |
| *Anisakis brevispiculata** |  | AB592803 |
| *Anisakis brevispiculata** |  | AB592805 |
| *Anisakis brevispiculata** |  | KC342899 |
| *Anisakis brevispiculata** |  | KJ786285 |
| *Anisakis ziphidarum** |  | KC821738 |
| *Anisakis ziphidarum** |  | KF214800 |
| *Anisakis ziphidarum** |  | KF214801 |
| *Anisakis ziphidarum** |  | KF214805 |
| *Contracaecum osculatum** |  | EF122211 |
| *Toxocara canis** |  | AF179923 |
